# MSF-Swin-ICFNet: An Image-Derived Multi-Scale Swin Transformer Framework with Cross-Scale Feature Fusion for Breast Lesion Classification and Segmentation in Mammograms

**DOI:** 10.3390/tomography12090130

**Published:** 2026-09-10

**Authors:** Narayanam R. S. Lakshmi Prasanthi, N. Thirupathi Rao, S. Deva Kumar

**Affiliations:** 1 Department of Computer Science and Engineering, Vignan’s Foundation for Science, Technology & Research (Deemed to be University), Vadlamudi, Guntur 522 213, AP, India; kprasanthi2025@gmail.com (N.R.S.L.P.); sdk_cse@vignan.ac.in (S.D.K.); 2 Department of Computer Science and Engineering, Vignan’s Institute of Information Technology(A), Visakhapatnam 530 049, AP, India

**Keywords:** breast cancer, mammography, Swin Transformer, image-derived feature fusion, cross-scale fusion, lesion classification, lesion segmentation, breast lesion localization, medical image analysis

## Abstract

**Background**: Accurate breast lesion classification and localization from mammograms remain challenging because lesions vary in size, morphology, density, contrast, and boundary clarity. This study proposes MSF-Swin-ICFNet, an image-derived Multi-Scale Swin Transformer framework with Image-Derived Cross-Scale Fusion (ICF) for joint benign–malignant classification and lightweight lesion localization. **Methods**: The proposed framework extracts hierarchical representations from four Swin Transformer stages and aligns, adaptively recalibrates, and fuses these multi-scale features into a shared representation for classification and lesion-mask prediction. The model was evaluated on CBIS-DDSM, comprising 891 patients and 1592 mammograms, and RTM, comprising 573 patients and 10,070 mammograms. Patient-level stratified partitioning was used to ensure that mammograms and associated annotations from the same patient remained within a single data partition. Classification was evaluated using accuracy, precision, recall, F1-score, and AUC-ROC, while lesion localization was assessed using the Dice coefficient. **Results**: MSF-Swin-ICFNet achieved strong mammogram-level classification performance on the patient-disjoint test cohorts. On CBIS-DDSM, the model achieved 0.949 accuracy, 0.931 F1-score, and 0.985 AUC-ROC. On RTM, it achieved 0.968 accuracy, 0.956 F1-score, and 0.992 AUC-ROC. The corresponding Dice coefficients for lesion localization were 0.786 on CBIS-DDSM and 0.878 on RTM. Ablation experiments further supported the contribution of cross-scale fusion, scale recalibration, feature refinement, and the boundary-aware localization head. **Conclusions**: MSF-Swin-ICFNet demonstrates promising retrospective performance for image-based breast lesion classification with complementary lesion localization. The results support the technical potential of adaptive hierarchical feature fusion for mammographic analysis. However, the model is not intended as a standalone diagnostic system, and independent multicentre validation and radiologist reader studies are required before clinical deployment.

## Simple Summary

Breast lesions can be difficult to distinguish on mammograms because they vary considerably in size, shape, density, contrast, and visibility. This study developed MSF-Swin-ICFNet, an artificial-intelligence model that examines mammograms at multiple image scales and combines information from these scales to classify breast lesions as benign or malignant while also indicating their approximate location. The model was evaluated using two mammography datasets, CBIS-DDSM and RTM, with patients separated between training and testing to reduce information leakage. It achieved classification accuracies of 94.9% on CBIS-DDSM and 96.8% on RTM and produced lesion-localization masks. These findings suggest that combining image information from multiple scales can support breast-lesion analysis using mammograms alone. However, the model has been evaluated retrospectively, and independent multicentre validation and radiologist reader studies are required before considering its use in clinical practice.

## 1. Introduction

Breast cancer remains one of the most common malignancies among women and is a major cause of cancer-related mortality worldwide. Early and accurate detection of breast lesions is therefore essential for improving treatment planning and patient outcomes. Mammography is widely used for breast cancer screening and diagnosis; however, interpretation remains challenging because lesion appearance varies with breast density, lesion morphology, contrast, and imaging quality.

Manual interpretation of mammograms is time-consuming and may be affected by inter-observer variability, especially when lesions are small, low-contrast, irregular, or obscured by dense breast tissue. These challenges have motivated the development of computer-aided diagnosis systems based on machine learning and deep learning [1,2]. Although convolutional neural networks have shown strong performance in medical image classification and segmentation, their local receptive fields may limit their ability to model long-range contextual relationships [3]. Transformer-based architectures, particularly Swin Transformers, provide an effective mechanism for hierarchical contextual representation, but they may require additional architectural support to preserve local boundary information [4,5]. Existing Swin Transformer-based segmentation frameworks commonly employ a U-shaped architecture in which hierarchical encoder representations are transferred to a comparatively large decoder through direct skip connections. Hybrid CNN–Transformer models usually employ a separate convolutional pathway to recover local texture and boundary information and subsequently combine it with transformer-derived contextual features through addition or concatenation. Although these approaches are effective, direct fusion may assign equal importance to feature levels with different semantic and spatial characteristics, while parallel encoders and full segmentation decoders increase architectural complexity.

To address these limitations, this study proposes MSF-Swin-ICFNet, an image-derived Swin Transformer framework with an explicit Image-Derived Cross-Scale Fusion (ICF) mechanism. The proposed fusion strategy differs from conventional Swin Transformer encoder–decoder architectures in which hierarchical encoder features are transferred directly to corresponding decoder stages through skip connections. It also differs from hybrid CNN–Transformer approaches that extract local and contextual representations through separate branches and subsequently combine them using fixed addition or concatenation [6]. In MSF-Swin-ICFNet, feature maps from all four Swin Transformer stages are first projected and spatially aligned to a common feature space, their relative contributions are adaptively recalibrated, and the resulting scale-weighted representations are fused into a shared image-derived feature representation [7,8,9,10,11]. Thus, the proposed strategy uses the complete hierarchical Swin feature pyramid without requiring a parallel CNN encoder or full symmetric segmentation decoder. The fused representation is subsequently shared by the benign–malignant classification head and the lightweight lesion-localization head.

Recent breast imaging studies have increasingly adopted transformer-based and hybrid CNN–Transformer models to combine local texture representation with global contextual modelling [12,13,14,15,16,17,18,19,20,21,22]. However, mammographic lesion analysis remains difficult because small masses, calcifications, irregular margins, and dense tissue patterns may appear at different spatial scales. Therefore, a model designed for mammography should preserve fine boundary cues while also learning broader contextual relationships across the image. The proposed MSF-Swin-ICFNet framework addresses this requirement through hierarchical Swin Transformer feature extraction, image-derived cross-scale fusion, lightweight feature refinement, and a boundary-aware mask head.

In the proposed framework, segmentation is used as a complementary lesion-localization output rather than as an independent diagnostic endpoint. The predicted mask provides spatial information about the image region associated with the model’s lesion representation and can therefore be overlaid on the original mammogram to indicate the approximate location and spatial extent of the suspected lesion. From an interpretability perspective, this output allows a radiologist to examine whether a benign–malignant classification is supported by a spatially plausible abnormal region rather than relying solely on the predicted probability. The localization map may also help identify discordant cases, for example when a high malignancy probability is produced without a clearly localized lesion or when the predicted region extends substantially beyond the visually suspicious area. In this way, the segmentation output may provide supplementary image-based evidence during a retrospective review and may support interpretation of the model’s classification decision. However, the generated masks are not intended to replace radiologist lesion delineation, BI-RADS assessment, or pathology-based diagnosis, and their value for clinical decision-making must be established through independent external validation and radiologist reader studies.

The primary contributions of this research are as follows:

### 1.1. Contributions

- An Image-Derived Cross-Scale Fusion mechanism that aligns and adaptively recalibrates four hierarchical Swin Transformer feature levels.
- A shared fused representation for benign–malignant classification and lightweight lesion localization without a parallel CNN encoder or full symmetric decoder.
- Component-wise evaluation of cross-scale fusion, scale recalibration, feature refinement and boundary-aware mask prediction
- Evaluation of a public benchmark and a clinically provided de-identified image collection, with the evaluation units and dataset limitations explicitly reported.
- Unlike SwinCAMF-Net, which performs multimodal fusion of mammographic, volumetric, and clinical information through cross-attention, the proposed MSF-Swin-ICFNet focuses on image-based hierarchical feature integration across multiple Swin stages. The proposed framework is therefore designed to improve cross-scale feature representation without requiring additional volumetric or clinical modalities.
- A direct architectural comparison is made with the previously published SwinCAMF-Net framework to establish that the present method addresses a distinct image-derived feature-fusion setting.

### 1.2. Novelty Aspects of the Proposed MSF-Swin-ICFNet Model

The scientific contribution of MSF-Swin-ICFNet does not arise merely from excluding clinical metadata or volumetric ROI inputs. Its principal technical contribution is the Image-Derived Cross-Scale Fusion (ICF) mechanism, which operates directly on the complete four-stage hierarchical feature pyramid generated by the Swin Transformer. Unlike approaches that use only the deepest representation, direct skip transfer, or fixed addition/concatenation of selected feature levels, ICF first transforms the four heterogeneous Swin representations into a common feature space, spatially aligns them, estimates the relative contribution of each scale, and performs adaptive scale-weighted fusion. Consequently, shallow representations containing comparatively fine lesion-boundary information and deeper representations containing higher-level semantic context contribute to the fused representation according to learned scale importance rather than being combined with equal predetermined weights.

A second distinguishing aspect is that the resulting fused representation is shared by two complementary tasks: benign–malignant classification and lightweight boundary-aware lesion localization. Thus, the framework does not require a parallel CNN feature extractor or a full symmetric encoder–decoder segmentation pathway. The technical novelty therefore lies in the combination of (i) complete four-stage hierarchical Swin feature utilization; (ii) explicit cross-scale alignment; (iii) adaptive scale recalibration prior to fusion; and (iv) shared use of the recalibrated fused representation for classification and lightweight localization. Removal of multimodal inputs should consequently be viewed as a difference in the intended input setting rather than as the primary source of novelty.

### 1.3. Relationship with Previous Work

The authors previously published a related article entitled “SwinCAMF-Net: Explainable Cross-Attention Multimodal Swin Network for Mammogram Analysis” in Diagnostics in 2025. That study proposed a multimodal framework integrating mammographic views, ROI-derived volume representations, and clinical metadata using a cross-attention fusion module. The present study is related to clinical problem setting and dataset usage, but it is architecturally different in scope and design. The present framework is an image-derived cross-scale fusion model that does not use clinical metadata, clinical projection, or volumetric ROI input. Its focus is to investigate whether multi-resolution mammographic image features alone can support breast lesion classification and segmentation through cross-scale feature integration and a lightweight mask head. Therefore, the present manuscript should be interpreted as a related but distinct image-derived extension, not as a duplicate of the earlier multimodal SwinCAMF-Net work. Table 1 presents the difference between the SwinCAMF-Net study and the present framework in the manuscript.

## 2. Literature Review

Breast cancer remains a major public health concern among women, and mammography is one of the most widely used imaging modalities for screening and early diagnosis. Despite its clinical value, mammogram interpretation is challenging because benign and malignant lesions may show overlapping features in shape, density, margin appearance, and contrast. Dense breast tissue, anatomical overlap, and subtle lesion boundaries further complicate automated detection and classification. As a result, to help radiologists find breast lesions more easily to classify and segment those lesions, computer-aided diagnosis (CAD) systems have been researched and developed extensively.

Publicly available mammography datasets have contributed significantly to the advancement of computer-aided diagnosis of breast cancer. The CBIS-DDSM is one of the most widely used benchmark datasets for the research of automated breast cancer diagnosis. CBIS-DDSM is a curated digital version of the Digital Database for Screening Mammography (DDSM) and contains mammograms of breast lesions with annotations and pathology-related data. While DDSM contains normal, benign, and malignant digital mammographic examinations, the CBIS-DDSM includes improved data curation, updated lesion segmentations, bounding boxes, and pathology codes, making the CBIS-DDSM data ideal for the development and evaluation of deep learning-based methods for the diagnosis of breast cancer.

Early CAD systems relied on handcrafted descriptors such as shape, texture, intensity, and margin features, followed by classifiers such as support vector machines, random forests, or k-nearest neighbours. Although these approaches provided diagnostic support, their performance depended heavily on manually designed features. As a result, their generalizability was limited when lesion appearance varied across scanners, acquisition settings, breast densities, and patient populations. Deep learning methods have become popular because they can automatically learn discriminative features from mammographic images without depending on manually designed descriptors.

Convolutional neural networks have been widely used for mammographic image analysis because they can learn local spatial patterns such as edges, texture, lesion margins, and density variations. Investigations have shown that CNN models such as EfficientNet, VGG, DenseNet and ResNet are effective in classifying mammograms and finding abnormalities, as the CNN models are capable of extracting multi-level, hierarchical features from images at varying levels of detail, starting from low-level texture features (e.g., edges) through to high-level semantic representations of the images [13,14,15]. However, due to the limited size of a CNN’s local receptive field, there are limits to the spatial features that can be learned to represent long-range contextual information. This fact becomes particularly important when considering how a radiologist would evaluate a mammogram with a possible lesion; in order to evaluate whether there is a lesion, it is essential to consider both the lesion itself and its immediate environment, including the normal composition of tissue surrounding the lesion, the symmetry of the breast, as well as the normal patterns of breast tissue.

U-Net is a widely used encoder–decoder architecture for biomedical image segmentation [3,7]. Its contracting path extracts contextual information, while its expanding path restores spatial resolution for pixel-level localization. Skip connections transfer encoder features to the decoder, which is particularly useful when precise lesion boundary delineation is required. The encoder and decoder components of U-Net include a contracting component, which serves to downsample the image and to capture thorough contextual information at a variety of scales, and a corresponding expanding component, which serves to upsample the image to restore the spatial resolution of the resulting segmented image, for exact localization of objects of interest (e.g., lesions). Additionally, U-Net uses skip connections to transfer spatial feature information learned during the compression (i.e., encoding) to the decompressed version (i.e., decoding) of the images. This ability to transfer spatial feature information is especially critical with respect to medical image segmentation applications where precise boundary delineation is required.

Several U-Net variants have been developed to improve mammogram segmentation performance. U-Net++ employs nested and dense skip connection-based pathways from the encoder to the decoder, which adds to the efficiency of representing semantic differences between the encoder and decoder feature sets [8]. Attention U-Net integrates attention gates into the existing U-Net architecture to direct the network’s attention to relevant target structures while diminishing the influence of irrelevant background regions [9]. Attention gates are important for analyzing small lesions, which are frequently present in the background of complex anatomical structures in clinical medical images. The effectiveness of using attention gates within the analysis of mammogram images to concentrate on target (i.e., lesion) regions with heterogeneous contour and size, eliminating the need for the inclusion of additional explicit localization modules within their analysis [5].

Despite the success of CNN- and U-Net-based methods, breast lesion analysis remains challenging because lesions may appear as irregular masses, diffuse density variations, architectural distortions, or small calcifications. These abnormalities occur at different spatial scales, and a single-scale feature extraction method may fail to capture both fine lesion boundaries and global contextual information. Therefore, multi-scale feature learning has become important in biomedical image analysis. Multi-scale networks combine feature representations from different resolution levels, improving both lesion localization and classification performance in mammograms, especially when lesion size and morphology vary considerably.

Vision Transformers are becoming increasingly popular for medical image analysis because they can model long-range dependencies using self-attention [10]. In comparison to CNNs, which mainly learn local patterns through convolutions, Transformers model relationships between distant image regions [11,12]. This ability is useful in mammography because diagnostic interpretation often requires understanding both the lesion region and the surrounding breast tissue context.

On the other hand, traditional Vision Transformers can be very computationally intensive and require large datasets, as the self-attention mechanism is applied to the entirety of the pixels in the image.

The Swin Transformer overcomes these challenges by implementing a hierarchical Transformer architecture with a shift-window approach. Instead of applying the global self-attention mechanism to all the pixels in the image (like traditional Vision Transformers), Swin Transformers utilize self-attention extracted locally from groups of windows that are shifted relative to the original [13]. This method allows for effective computation while also allowing for the extraction of multiple scales of representations. The Swin Transformer can be employed as the backbone for a variety of vision tasks, such as classification, detection, and segmentation. This hierarchical design supports efficient feature extraction at multiple resolutions and is therefore suitable for high-resolution medical images such as mammograms [14].

Many researchers have examined Swin Transformer-based segmentation and classification models for medical imaging. Researchers have found that Swin Transformers provide a viable pathway to achieve hierarchical feature learning through strong contextual modeling, due to the self-attention mechanism. The shift-window approach of the Swin Transformer is advantageous in terms of computational feasibility when applying global attention to high-resolution medical images, especially mammography. Due to the potential to lose local features when applying a pure transformer model, hybrid models that utilize both CNNs and transformers have gained popularity [14]. This approach combines the strength of the CNN for extracting local features and the strength of the Transformer to model long-range relationships, using self-attention.

Hybrids are particularly applicable to mammographic images. The CNN component provides the ability to extract local features such as texture of the input, edge detail of the input, and margins of lesions, while the transformer component extracts more abstract relationships and semantics of the input. Recent studies examining the segmentation of medical images using the hybrid CNN–Transformer approach have found that this model produces improved representation learning and segmentation performance, especially for data with lesion boundary ambiguity, low contrast, and complex anatomical backgrounds [15,16,17].

Attention-based feature fusion is the other major research direction. Features are typically extracted from many different levels in medical imaging networks, but they are frequently fused using simple addition or concatenation methods. These fusion techniques are computationally cheap but do not allow models to select the most relevant features dynamically. Attention-based fusion techniques and cross-attention mechanisms allow a model to give more weight to diagnostically significant features and reduce the weight assigned to features that are not relevant or are redundant. This is particularly advantageous for breast lesion analyses, as the abnormal areas may be small, difficult to see, and surrounded by significant normal tissue.

Despite the reported advances, the principal model categories involve different trade-offs. CNN and U-Net-based networks effectively preserve local texture and lesion-margin information, but their contextual modelling is constrained by convolutional receptive fields. Pure transformer and Swin Transformer models capture long-range relationships more effectively; however, successive patch merging and spatial downsampling may weaken small-lesion and boundary information. U-shaped transformer models recover spatial detail using decoders and skip connections, but these structures may increase computational demand and transfer redundant low-level features.

Hybrid CNN–Transformer architectures combine local convolutional features with global transformer representations, but many require parallel feature-extraction branches and employ fixed addition or concatenation during fusion. These operations do not explicitly determine whether a particular spatial scale is relevant to a given lesion. Attention-based fusion can partially address this issue, although some methods integrate only selected feature levels or use a full encoder–decoder pathway designed principally for segmentation. Consequently, there remains scope for a framework that adaptively integrates the complete hierarchical Swin feature pyramid and uses the fused representation for both classification and lightweight lesion localization. Table 2 presents the mammographic and medical-image analysis strategies.

From an architectural perspective, the proposed ICF mechanism differs from commonly used multi-scale integration strategies in three respects. First, conventional U-Net-type skip fusion transfers encoder representations directly to corresponding decoder levels, whereas ICF aligns all four hierarchical Swin stages into a common feature space before fusion. Second, hybrid CNN–Transformer approaches frequently combine independently extracted local and contextual representations through fixed addition or concatenation; in contrast, ICF operates entirely within the hierarchical Swin feature pyramid and recalibrates the contribution of each scale before aggregation. Third, attention-based multi-scale methods can dynamically emphasize informative representations, but several existing approaches operate on selected feature levels or remain embedded within comparatively large encoder–decoder segmentation structures. MSF-Swin-ICFNet instead uses adaptive four-stage cross-scale fusion to generate one shared image-derived representation that supports both classification and lightweight lesion localization. Therefore, the distinction lies not in multi-scale processing alone, but in the sequence of complete hierarchical feature extraction, explicit alignment, adaptive-scale recalibration, weighted fusion, and shared dual-task utilization.

Recent transformer-based breast-imaging studies can be differentiated according to prediction task, input organization, feature-integration strategy, and computational design, the author proposed a multi-stage framework for breast-lesion segmentation and classification [14], while author also investigated transformer-based explainable breast-lesion segmentation [15]. The authors had examined multi-view graph- and transformer-based architectures for mammography classification, and Incorporated shape-guided information into transformer-based breast-cancer prediction [16,17]. The authors had proposed Swin-DAMFN, in which a Swin Transformer branch for global-context modelling is combined with a CNN-based dual-attention multi-scale fusion branch for local mammographic features [18,19,20,21,22]. This architecture demonstrates the value of multi-scale global–local fusion but differs from the present framework because it employs parallel Transformer and CNN pathways rather than adaptive fusion of the complete hierarchical Swin feature pyramid.

Recent segmentation-oriented studies provide additional relevant comparisons. HTU-Net for breast-mass segmentation in mammograms, combining convolutional local representations with Transformer-based global context and a Multi-Scale Cross-Attention Transformer module within an encoder–decoder architecture [24]. In contrast, the proposed ICF mechanism does not use a parallel CNN–Transformer representation or conventional encoder–decoder skip fusion; instead, all four Swin stages are projected and aligned into a common feature space before adaptive scale weighting. The authors had introduced a multi-scale attention-based Swin Transformer architecture for medical-image segmentation, demonstrating the broader relevance of efficient hierarchical and multi-scale attention mechanisms, although that framework is segmentation-oriented and was not developed specifically for mammographic joint classification and localization [25].

More recent mammography-specific Swin architectures further demonstrate the rapid development of this area. LAM-CATNet, a Lambda-aware multi-scale cross-attention Swin framework combining Transformer-derived context with U-Net-based local representations for mammographic lesion segmentation and classification [26]. The authors had investigated a Swin-based framework for analysis of individual mammograms using hierarchical feature learning and optimization of Swin hyperparameters [27]. These studies confirm that Swin-based hierarchical representation, multi-scale integration, and single-mammogram analysis are active research directions. Therefore, the novelty of MSF-Swin-ICFNet should not be attributed simply to the use of Swin Transformer, multi-scale features, or image-only input. Its specific architectural contribution is the explicit alignment of the complete four-stage Swin hierarchy into a common feature space, adaptive estimation of scale contributions, weighted cross-scale fusion, and use of the resulting shared representation for both benign–malignant classification and lightweight lesion localization without a separate CNN encoder or full U-shaped segmentation decoder [28].

Because the recent methods differ in dataset composition, prediction endpoint, input organization, architecture, and evaluation protocol, direct numerical ranking across published studies would not constitute a controlled comparison. The present literature comparison is therefore used to establish architectural similarities and differences, while quantitative model comparisons in this study are restricted to architectures reimplemented under the common patient-disjoint experimental protocol.

Overall, the literature does not indicate a single architecture that simultaneously optimizes local boundary preservation, long-range contextual modelling, architectural simplicity, input availability, and joint classification–localization capability. CNN/U-Net approaches retain strong spatial detail but provide comparatively limited long-range modelling; pure Transformer approaches strengthen contextual representation but may weaken fine spatial information through hierarchical downsampling; hybrid CNN–Transformer and attention-based methods improve feature complementarity but may introduce additional branches or fusion complexity; and multimodal approaches can exploit patient-specific information but depend on the consistent availability of additional clinical inputs. These trade-offs motivate the present investigation of an image-derived architecture that adaptively integrates hierarchical Swin features while using a shared representation for classification and lightweight lesion localization.

The proposed design is intended to improve global context modelling while preserving local spatial information from mammographic images. Unlike multimodal or volumetric approaches, the present framework relies only on image-derived Swin Transformer features. The Image-Derived Cross-Scale Fusion module integrates multi-resolution feature maps, while the lightweight boundary-aware mask head supports lesion localization. Therefore, MSF-Swin-ICFNet provides an image-only framework for breast lesion classification and segmentation using CBIS-DDSM and RTM datasets.

A related previous study was proposed SwinCAMF-Net, an explainable multimodal Swin Transformer framework for mammogram analysis [23]. That model integrated mammographic views, ROI-derived representations and clinical metadata using a multimodal cross-attention fusion module for joint classification and segmentation. While that study demonstrated the value of multimodal fusion, its architecture depends on additional non-image or volumetric information streams. In contrast, the present study focuses on a proposed framework that uses only mammographic image feature representations, thereby addressing a different practical scenario where clinical metadata or ROI-derived inputs may not be consistently available.

Multimodal learning represents an important complementary direction in which imaging-derived representations are integrated with patient-level clinical variables. Such integration can be advantageous when clinical descriptors provide information that is not fully encoded in the image itself. For example, Combined CT-derived deep-learning features with clinical descriptors for predicting pembrolizumab response in advanced non-small-cell lung cancer [23]. Their patient-level analysis showed that the combined clinical + deep-learning configuration provided better balanced predictive performance than either the deep-learning-only or clinical-only configurations, illustrating that clinical variables can contribute complementary information to image-derived representations. Although the disease, imaging modality, cohort size, and prediction endpoint differ substantially from mammographic lesion classification, the study demonstrates that exclusion of clinical information should not itself be interpreted as an architectural or predictive advantage.

Accordingly, the present study does not argue that image-only modelling is inherently superior to multimodal modelling. Rather, image-only and multimodal approaches address different methodological and deployment settings. Multimodal systems may exploit complementary patient-specific information and potentially improve individualized prediction when sufficiently complete, reliable, and standardized clinical variables are available. Conversely, their performance and portability may be affected by missing variables, heterogeneous recording practices, and differences in the availability or definition of clinical descriptors across institutions. The objective of MSF-Swin-ICFNet is therefore not to replace multimodal approaches, but to investigate a distinct image-derived representation problem: whether the complete hierarchical Swin feature pyramid can be explicitly aligned, adaptively recalibrated, and jointly exploited for classification and lightweight lesion localization. A direct image-only versus image–clinical comparison using identical mammography cohorts, patient partitions, and standardized clinical variables would be valuable future work.

### 2.1. Research Gap

Despite advances in CNN-, Transformer-, hybrid, and multimodal frameworks for medical-image analysis, an unresolved architectural problem remains in efficiently exploiting the complementary information contained across the complete hierarchical feature pyramid of a Swin Transformer. Shallow stages retain comparatively fine spatial and lesion-boundary information, whereas deeper stages encode progressively more abstract semantic and contextual representations. Existing approaches commonly use the deepest representation, transfer encoder features through decoder skip connections, combine selected scales using fixed addition or concatenation, or employ separate CNN and Transformer branches. Although attention-based fusion can improve feature selection, not all approaches explicitly align the complete four-stage Swin hierarchy and learn the relative contribution of each scale before constructing a shared representation for both classification and lesion localization. Therefore, the primary research gap addressed in this study is not the absence of clinical metadata. Rather, it is the lack of an explicitly formulated image-derived mechanism that performs complete hierarchical feature alignment, adaptive cross-scale recalibration, weighted fusion, and shared utilization of the resulting representation for benign–malignant classification and lightweight lesion localization. The image-only setting defines the scope of the present investigation, while multimodal image–clinical fusion remains a complementary research direction when appropriate clinical information is available.

### 2.2. Motivation

The study is motivated by the complementary information encoded at different stages of a hierarchical Swin Transformer. Mammographic lesions may contain fine margin, texture, and boundary characteristics together with broader contextual patterns in the surrounding breast tissue, and these characteristics are represented differently across shallow and deep feature levels. An effective fusion mechanism should therefore preserve the contribution of fine spatial representations while also exploiting deeper semantic context and should determine the relative importance of these feature levels adaptively rather than combining them with predetermined equal weights. MSF-Swin-ICFNet was developed to investigate this image-representation problem through cross-scale alignment, adaptive recalibration, and shared classification–localization learning. Its image-only configuration represents the scope of the present experiment rather than a claim that clinical variables lack diagnostic value.

### 2.3. Proposed Work

This study proposes MSF-Swin-ICFNet, an image-derived framework consisting of hierarchical Swin Transformer feature extraction, adaptive cross-scale feature fusion, a benign–malignant classification head, and a lightweight lesion-localization head. The framework is evaluated using the CBIS-DDSM and RTM mammography datasets through classification, segmentation, patient-grouped cross-validation, computational assessment, comparative experiments, and ablation analysis.

### 2.4. Objectives of the Work

The primary objective of this work is to investigate whether adaptively fused hierarchical mammographic image features can support reliable benign–malignant classification together with lesion localization in an image-only framework. A secondary objective is to evaluate the proposed design on CBIS-DDSM and RTM using classification and segmentation measures, patient-grouped cross-validation, computational analysis, comparisons with reimplemented architectures, and component-wise ablation experiments.

## 3. Materials and Methods

### 3.1. Data Collection

To evaluate the proposed model, two mammography datasets were considered: the publicly available CBIS-DDSM benchmark and the retrospective de-identified RTM clinical mammography cohort [2]. The RTM dataset was provided for research through the Department of Radiation Oncology, HIMS Medical College, Government of Karnataka, India, and represents patients primarily from Karnataka and adjoining border regions of Andhra Pradesh. Following de-identification and transfer for research use, the dataset was maintained at the MIC Lab, Vignan’s Institute of Information Technology, Duvvada, Visakhapatnam, Andhra Pradesh, India. The inclusion of these two cohorts enabled evaluation using both a curated public benchmark and a clinically acquired mammography collection with different dataset characteristics.

The proposed network accepts and processes one mammographic image at a time. Therefore, the primary analysis reported in the main manuscript is conducted at the mammogram level. Nevertheless, dataset partitioning was performed using patient identifiers to ensure that all mammograms, views, lesion annotations, masks, and derived samples belonging to the same patient remained within one training, validation, or testing partition. Complete patient-level cohort statistics and train–validation–test partition counts are provided in Supplementary Table S1, while leakage-verification and cross-validation configuration details are provided in Supplementary Tables S2 and S3.

#### 3.1.1. CBIS-DDSM Dataset

We utilized the Curated Breast Imaging Subset of DDSM (CBIS-DDSM) [2] as our main benchmark dataset, which consists of digitally imaged mammographic images with breast abnormalities, including cancers, masses and calcifications. There is a wealth of expert annotations, pathology diagnostic labels based on pathology, and lesion-level data pertaining to each image; therefore, this dataset can be used for both breast lesion classification and segmentation tasks. Each image is in DICOM format and contains standard mammographic views to provide detailed information on the benign or malignant nature of the breast findings. The model processed each CBIS-DDSM mammogram independently. The available case identifiers were used only to construct mutually exclusive patient groups before model development; they were not used as predictive inputs. Detailed patient counts, diagnostic distributions, and partition composition are reported in Supplementary Table S1. For CBIS-DDSM, the lesion-level annotations and diagnostic labels were used as provided with the curated dataset. The lesion annotations define the spatial regions associated with the corresponding mammographic findings, while the benign/malignant diagnostic labels are linked to the available pathology-based case information. These dataset-provided annotations were used as reference targets for the lesion-localization task and were not manually redrawn or modified by the authors. The present study therefore evaluates the model against the released CBIS-DDSM reference annotations rather than introducing a new independent annotation protocol.

#### 3.1.2. RTM Dataset

The RTM dataset represents a retrospective, de-identified clinical mammography cohort provided for research through the Department of Radiation Oncology, HIMS Medical College, Government of Karnataka, India. The patient cohort primarily represents individuals from Karnataka and the adjoining border regions of Andhra Pradesh. Following de-identification and transfer for research use, the dataset was maintained at the MIC Lab, Vignan’s Institute of Information Technology, Duvvada, Visakhapatnam, Andhra Pradesh, India. The dataset was used under institutional data-sharing permission, and no directly identifiable patient information was used in the present analysis.

The RTM dataset comprised 573 unique patients and 10,070 de-identified mammograms. The cohort included 307 patients in the benign category, contributing 5374 mammograms, and 266 patients in the malignant category, contributing 4696 mammograms. All mammograms associated with an individual patient were grouped using the corresponding patient identifier before data partitioning. Thus, patient identity was used only for leakage-controlled partitioning and was not supplied to the model as a predictive feature.

A stratified 70:15:15 patient-level split with a fixed random seed of 42 was used. This resulted in 401 training patients, 86 validation patients, and 86 independent test patients. The training partition contained 215 benign and 186 malignant patients and comprised 7083 mammograms. The validation partition contained 46 benign and 40 malignant patients and comprised 1498 mammograms. The independent test partition contained 46 benign and 40 malignant patients and comprised 1489 mammograms. All mammograms and available lesion annotations belonging to a given patient were retained within the same partition.

For the RTM cohort, lesion masks were supplied by the dataset provider and were used as the reference annotations for model training and segmentation evaluation. The accompanying dataset documentation available to the authors did not provide sufficient information regarding the annotation workflow, including whether the boundaries were generated manually or semi-automatically, the number and professional qualifications of annotators, the use of independent double reading, inter-observer agreement, or consensus review. Accordingly, the present study does not claim that the RTM masks represent independently verified expert consensus annotations. They are treated as provider-supplied reference masks, and this limitation is considered when interpreting the segmentation results.

Detailed and harmonized information regarding mammography scanner vendor and model, detector characteristics, exposure parameters, acquisition protocol, patient age distribution, ethnicity, menopausal status, and breast-density distribution was not available in the dataset documentation used for the present analysis. Consequently, equipment-specific, demographic, and breast-density subgroup analyses could not be performed. The geographical composition of the cohort should also be considered when interpreting generalizability because the available patients were predominantly drawn from Karnataka and adjoining border regions of Andhra Pradesh rather than from a geographically diverse multicentre population.

The RTM lesion masks were supplied as part of the dataset and were treated as provider-supplied reference annotations. The available documentation did not specify whether lesion boundaries were generated manually, semi-automatically, or through a consensus procedure. Information regarding the number of annotators, their professional qualifications, radiological specialization, years of experience, inter-observer agreement, and consensus-review procedure was also unavailable. Accordingly, the RTM masks are not characterized in the present study as independently verified expert clinical ground-truth delineations.

All mammograms, lesion masks, annotations, and derived samples belonging to the same patient were grouped using the anonymous patient identifier before dataset partitioning. This grouping ensured that data originating from one patient were retained within a single training, validation, or testing partition and were not independently distributed across model-development and evaluation subsets.

#### 3.1.3. Patient-Level Partitioning and Leakage Control

Dataset partitioning was performed at the patient level before preprocessing-dependent augmentation and model training. All mammograms, standard views, lesion annotations, masks, lesion-derived samples, and other derived data associated with the same canonical patient identifier were first grouped together. A diagnostic class was assigned at the patient level, after which patients were stratified according to benign–malignant status and allocated to training, validation, and testing cohorts using a 70:15:15 ratio and a fixed random seed of 42. Following patient assignment, every mammogram and corresponding annotation belonging to that patient inherited the same partition label. Images were therefore never independently randomized across the three subsets.

For CBIS-DDSM, this procedure resulted in 624 training patients, 133 validation patients, and 134 test patients, corresponding to 1114, 239 and 239 full mammograms, respectively. The training cohort contained 301 benign and 323 malignant patients; the validation cohort contained 65 benign and 68 malignant patients; and the test cohort contained 65 benign and 69 malignant patients.

The same patient-grouping principle was applied to the RTM cohort. All mammograms and associated lesion masks belonging to a patient were required to remain within the same partition. The finalized revised RTM training, validation, and test counts are reported in Table 3 and the Supplementary Materials.

Data augmentation was performed only after patient-level partitioning and was restricted to the training cohort. Validation and test mammograms were not augmented. The segmentation subset was not independently repartitioned; mask-bearing mammograms inherited the partition of their corresponding source patient. For grouped cross-validation, complete patient groups were assigned to folds while the independent test cohort remained excluded from model development. Patient identifiers were audited across partitions to identify duplicate, missing, or cross-partition identifiers.

### 3.2. Data Preprocessing

A standardized preprocessing pipeline was applied to all mammographic images before model training. Each image was resized to 512 × 512 pixels while preserving the original aspect ratio using padding where necessary. Noise suppression was performed using a Wiener filter with a 3 × 3 window to reduce acquisition-related noise while preserving lesion edges. Contrast Limited Adaptive Histogram Equalization (CLAHE) was applied with a clip limit of 2.0 and an 8 × 8 grid size to enhance low-contrast lesion regions. The breast region was extracted using Otsu thresholding followed by morphological operations to remove labels, background regions, and non-breast artifacts. Finally, pixel intensity values were normalized to the range of 0–1 to provide consistent input to the proposed MSF-Swin-ICFNet framework.

### 3.3. Data Normalization

Following spatial preprocessing and breast-region extraction, intensity normalization was applied to reduce inter-image and inter-source intensity variability. First, the pixel intensities were scaled to the range [0,1] using min–max normalization. The normalized images were subsequently standardized using z-score normalization, with the mean and standard deviation computed according to the predefined normalization protocol used during model development. Histogram matching was additionally applied using a fixed reference intensity distribution to reduce systematic differences in intensity distributions between mammographic images acquired under different imaging conditions. Where tissue-aware normalization was used, the normalization was applied within the extracted breast region to reduce intensity variation between dense and relatively fatty tissue regions while preserving lesion contrast. The same normalization procedure was applied to the training, validation, and test images without using diagnostic labels or test-set information to estimate normalization parameters. Consequently, normalization was treated as an image-processing operation and not as a source of label-dependent information.

### 3.4. Data Augmentation

Many medical imaging datasets are small and may have class imbalance, especially for malignant cases and small lesions. To improve generalization of the model and reduce the likelihood of overfitting, data augmentation was used during training by implementing an augmentation pipeline that included geometric, intensity-based, noise-based, and lesion-aware augmentations. Geometric augmentation included random rotations to ±15 degrees, horizontal flips, random translations of ±10%, and random scaling between 0.9 and 1.1 to mimic the natural variations in positioning of the patient’s breast and the way that images are taken during a mammogram. Intensity augmentation included adjustments made to brightness and contrast within ±10%, and adjustments to gamma between 0.8 and 1.2, to account for different contrasts and amounts of exposure throughout mammographic imaging. Gaussian noise (σ = 0.01) and Gaussian blur (σ = 0.5) were used as augmentation techniques to help develop robustness against the artifacts that result from acquisition. Mixup augmentation was performed by creating linear combinations of all image pairs with sampled mixing weights taken from a beta distribution using α = 0.2. Lesion-aware augmentation was restricted to training mammograms for which lesion annotations were available. Lesion locations were obtained from the reference lesion masks supplied with the corresponding dataset; a separate lesion-detection model was not used to identify the augmentation target. The lesion masks were used to identify the spatial extent of annotated lesions and to preferentially sample mammograms containing small or under-represented lesion patterns. For geometric augmentations, the same spatial transformation was applied synchronously to the mammogram and its corresponding lesion mask so that pixel-level alignment was preserved. Lesion-aware augmentation was performed only within the training partition and did not alter the validation or test cohorts. Further leakage-control details are provided in Supplementary Table S2. For lesion-aware augmentation, the reference lesion mask was used to determine the spatial extent of the annotated lesion before transformation. Training samples containing relatively small or under-represented lesions were preferentially selected for lesion-aware augmentation. The selected mammogram and its corresponding mask were treated as a paired sample. Any geometric transformation applied to the mammogram, including rotation, translation, scaling, or flipping, was applied using the identical transformation parameters to the corresponding lesion mask. Intensity-only transformations were applied to the mammographic image and did not modify the binary mask. Consequently, the lesion boundary remained spatially aligned with the transformed mammogram throughout augmentation. No lesion mask was used to modify, generate, or augment validation or test images. The reference masks were used only to identify the lesion region for training-time lesion-aware augmentation and to provide the target masks for the segmentation objective. The datasets characteristics and patient level partitioning details are presented in Table 4. The augmentation details are presented in Table 5.

### 3.5. Image-Derived Feature Extraction

The proposed MSF-Swin-ICFNet model employs an image-derived hierarchical feature extraction strategy to capture both local lesion characteristics and global contextual information from mammographic images. Each preprocessed mammogram of size (512 × 512) pixels is partitioned into non-overlapping (4 × 4) patches. The resulting patches are projected into a 96-dimensional embedding space, producing an initial feature representation of (128 × 128 × 96).

The embedded features are processed through four hierarchical stages of the Swin Transformer encoder. The first stage operates on the (128 × 128) feature representation with 96 channels. Successive patch-merging operations reduce the spatial resolution by a factor of two while increasing the feature dimension, resulting in feature representations of (64 × 64 × 192), (32 × 32 × 384) and (16 × 16 × 768) at stages 2, 3, and 4, respectively. Thus, the four hierarchical representations correspond to spatial scales of (1/4), (1/8), (1/16), and (1/32) relative to the original (512 × 512) mammogram.

The hierarchical design enables the early stages to preserve comparatively fine lesion and boundary information, while the deeper stages provide increasingly abstract semantic and contextual representations. Feature maps from all four stages are retained and subsequently supplied to the Image-Derived Cross-Scale Fusion module for alignment, recalibration, and fusion.

Unlike the previous SwinCAMF-Net framework, the present model does not use clinical metadata, clinical projection, or external volumetric ROI input. Instead, the extracted Swin feature maps are treated as image-derived representations and passed to the Image-Derived Cross-Scale Fusion model, where multi-resolution feature maps are aligned and fused for classification and mask prediction.

Clarification regarding three-dimensional convolutions. MSF-Swin-ICFNet is a two-dimensional mammographic image model and does not employ 3D convolutions at any stage. Each input is a single 2D mammographic image represented as a 512 × 512 × 1 tensor. The four Swin Transformer stages generate hierarchical 2D feature maps, and the subsequent Image-Derived Cross-Scale Fusion and mask-refinement operations are also performed on 2D spatial feature maps. The 3D convolutional/volumetric representation belongs to the previously published SwinCAMF-Net architecture and is not part of the present MSF-Swin-ICFNet implementation. This distinction is important because the present study intentionally investigates image-derived 2D feature fusion without external volumetric ROI inputs. Table 6 provides the feature extraction strategy used in the proposed model.

### 3.6. Lightweight Cross-Scale Mask Head

The segmentation component of the current framework is implemented as a lightweight cross-scale mask head rather than a full multimodal encoder–decoder pathway. The fused image-derived features generated by the Cross-Scale Fusion module are progressively refined through convolutional refinement layers and upsampling operations to produce lesion localization masks. This design is intended to preserve lesion boundary information while avoiding the multimodal decoder structure used in the previous SwinCAMF-Net study. All convolutional operations in the proposed mask-refinement pathway are two-dimensional and operate on spatial feature maps of the form H × W × C. No three-dimensional convolution is used in MSF-Swin-ICFNet because the framework processes individual 2D mammograms and does not construct volumetric image representations. In addition, the mask head does not employ U-Net-style encoder-to-decoder skip connections. Instead, hierarchical information from the four Swin Transformer stages is integrated explicitly within the Image-Derived Cross-Scale Fusion module before being supplied to the refinement and localization pathway. Thus, cross-scale fusion replaces direct encoder–decoder skip transfer as the mechanism for combining information from different hierarchical resolutions.

Clarification regarding skip connections. The proposed mask head does not use conventional U-Net-style encoder-to-decoder skip connections. In a U-Net-type architecture, feature maps from corresponding encoder levels are directly transferred to decoder levels to restore spatial detail. In MSF-Swin-ICFNet, information from the four hierarchical Swin stages is instead projected to a common channel dimension and spatial resolution, adaptively recalibrated, and fused by the Image-Derived Cross-Scale Fusion module before being passed to the lightweight mask head. Thus, cross-scale fusion serves as the mechanism for integrating hierarchical information, rather than direct encoder-to-decoder skip transfer. The mask head subsequently performs two-dimensional convolutional refinement and bilinear upsampling to generate the lesion-localization mask.

The mask head receives the fused multi-scale representation and applies boundary-aware refinement to emphasize lesion margins while suppressing irrelevant background responses. No auxiliary intermediate mask supervision was used in the reported experiments. The final predicted mask was optimized against the available lesion annotation using binary cross-entropy and Dice losses. This lightweight formulation supports lesion localization while maintaining the image-only scope of the framework. Table 7 provides the segmentation details used in the proposed model.

### 3.7. Mathematical Formulation of Image-Derived Cross-Scale Fusion, Feature Refinement, and Loss Functions

Let I denote an input mammogram and let the four hierarchical stages of the Swin Transformer encoder be denoted by E_s_(⋅), s∈{1,2,3,4}. The corresponding feature representations are

F_s_ = E_s_ (I), s = 1,2,3,4. (1)

Because Fs differs across stages in both spatial resolution and channel dimension, each representation is transformed into a common fusion space. Let Ps (⋅) denote the stage-specific channel projection Rs (⋅), and the corresponding spatial-alignment operation. The aligned feature representation is

A_s_ = R_s_ (P_s_ (F_s_)), A_s_ ∈ R^H^_f_^×W^_f_^×C^_f_. (2)

Thus, all four hierarchical feature representations have identical spatial and channel dimensions before cross-scale fusion.

**Adaptive Cross-Scale Recalibration**

Although the aligned feature maps have a common dimensionality, their relative importance for a given mammogram may differ. Therefore, the proposed Image-Derived Cross-Scale Fusion (ICF) mechanism estimates a learnable relevance score for each hierarchical feature level. Let G_s_ (⋅;θ_s_) denote the learnable scale-scoring function for stage. The corresponding relevance score is

q_s_ = G_s_ (A_s_; θ_s_),s = 1,2,3,4, (3)

where q_s_ represents the learned relevance score of the s-th hierarchical feature level.

The four relevance scores are normalized using a softmax function to obtain adaptive scale-contribution coefficients:

(4) $$\alpha s=\frac{exp(q_{s})}{\sum_{k=1}^{4}exp(q_{k})},\;\sum_{s=1}^{4}\alpha s=1$$

The normalized coefficients satisfy

$$\sum_{s=1}^{4}\alpha s=1,\;0<\alpha s<1$$

Each aligned feature representation is then recalibrated according to its learned contribution:

F_s_ = α_s_ A_s_. (5)

The final Image-Derived Cross-Scale Fusion representation is obtained by aggregating the four recalibrated hierarchical representations:

(6) $$F_{\mathrm{ICF}}\;=\;\sum_{s=1}^{4}Fs=\;\sum_{s=1}^{4}\alpha sAs.$$

Equivalently,

F_ICF_ = α_1_ A_1_ + α_2_ A_2_ + α_3_ A_3_ + α_4_ A_4_. (7)

Unlike direct addition or fixed concatenation, this formulation allows the contribution of each Swin feature level to vary adaptively according to the input mammogram. Consequently, shallow representations containing comparatively fine spatial and boundary information and deeper representations containing higher-level semantic context can contribute to the fused representation according to their learned relevance.

**Feature Refinement and Lesion-Localization Pathway**

The fused representation F_ICF_ is shared by the classification and lesion-localization pathways. For localization, it is passed through the lightweight two-stage refinement head described in Table 5. The initial representation of the refinement pathway is

H_0_ = F_ICF_. (8)

For refinement stage l, the convolutional feature transformation is defined as

R_l_ = C_l_ (Hl − 1), l = 1,2, (9)

where C_l_ (.) denotes the learnable two-dimensional convolutional refinement operation.

The refined representation is subsequently spatially upsampled:

H_l_ = U_l_ (R_l_), l = 1,2, (10)

where U_l_ (.), denotes the corresponding upsampling operation.

Combining the refinement and upsampling operations gives

H_l_ = U_l_ (C_l_ (H_l−1_)), l = 1,2. (11)

In the implemented mask head, the refinement pathway uses two-dimensional convolutions with progressive channel refinement from 96 to 48 channels and bilinear upsampling, as specified in Table 5.

After the final refinement stage, the lesion probability map is generated using the output prediction layer:

M = σ(C_out_ (H_2_)), (12)

where C_out_(.) denotes the final one-channel mask-prediction operation, σ(.) denotes the sigmoid activation function, and

M ∈ [0,1]^H×W^ (13)

represents the predicted lesion-probability map.

The complete lesion-localization pathway can therefore be summarized as

FICF → H1 → H2 → M.

This formulation converts the fused hierarchical representation into a high-resolution lesion-localization output without employing three-dimensional convolution or U-Net-style encoder-to-decoder skip connections.

**Classification Loss**

For benign–malignant classification, let y_i_ ∈ {0,1} denote the ground-truth diagnostic label of the i-th mammogram and let p_i_ ∈ [0,1] denote the corresponding predicted probability of malignancy. The binary cross-entropy classification loss is defined as

(14) $$L_{\mathrm{cls}}=-\frac{1}{N}\sum_{i=1}^{N}\left[yi,\log\left(Pi\right)+\left(1-Yi\right).log(1-Pi)\right]$$

where N denotes the number of mammograms in the training batch.

**Segmentation Loss**

For lesion segmentation, let y_j_ ∈ {0,1} denote the reference value and y_j_ ∈ [0,1] denote the predicted probability for pixel j. The pixel-wise binary cross-entropy loss is

(15) $$L_{\mathrm{BCE}}=-\frac{1}{M}\sum_{i=1}^{M}\left[yj,\log\left(yj\right)+\left(1-Yj\right).log(1-yj)\right]$$

where M denotes the total number of pixels in the lesion mask.

(16) $$D=\frac{2\sum_{j=1}^{M}Yj+E}{\sum_{j=1}^{M}Yj}$$

where is a small positive constant introduced for numerical stability.

The corresponding Dice loss is

L_Dice_ = 1 − D (17)

The segmentation objective combines pixel-wise binary cross-entropy and region-overlap losses:

Lseg = λ_BCE_ L_BCE_ + λ_Dice_ L_Dice_, (18)

where λ_BCE_ and L_Dice_ control the relative contribution of the two segmentation-loss components.

Consequently, mammograms with lesion annotations contribute to both classification and segmentation optimization, whereas mammograms without lesion masks contribute only to the classification objective.

## 4. Proposed Model

The proposed MSF-Swin-ICFNet architecture is designed as an image-derived framework for breast lesion classification and segmentation from mammographic images. The model does not use clinical metadata, clinical projection modules, or external 3D ROI/volume inputs. Instead, it learns hierarchical mammographic representations through a Swin Transformer encoder and integrates multi-resolution image-derived features through a proposed framework.

The input mammogram is preprocessed and divided into image patches, which are processed by the Swin Transformer encoder to generate feature maps at multiple spatial resolutions. The proposed model aligns and combines these multi-level features so that low-level boundary information and high-level semantic context are jointly represented. The fused feature representation is passed to two task-specific heads: a classification head for benign/malignant prediction and a lightweight mask head for lesion localization.

**Figure 1:**
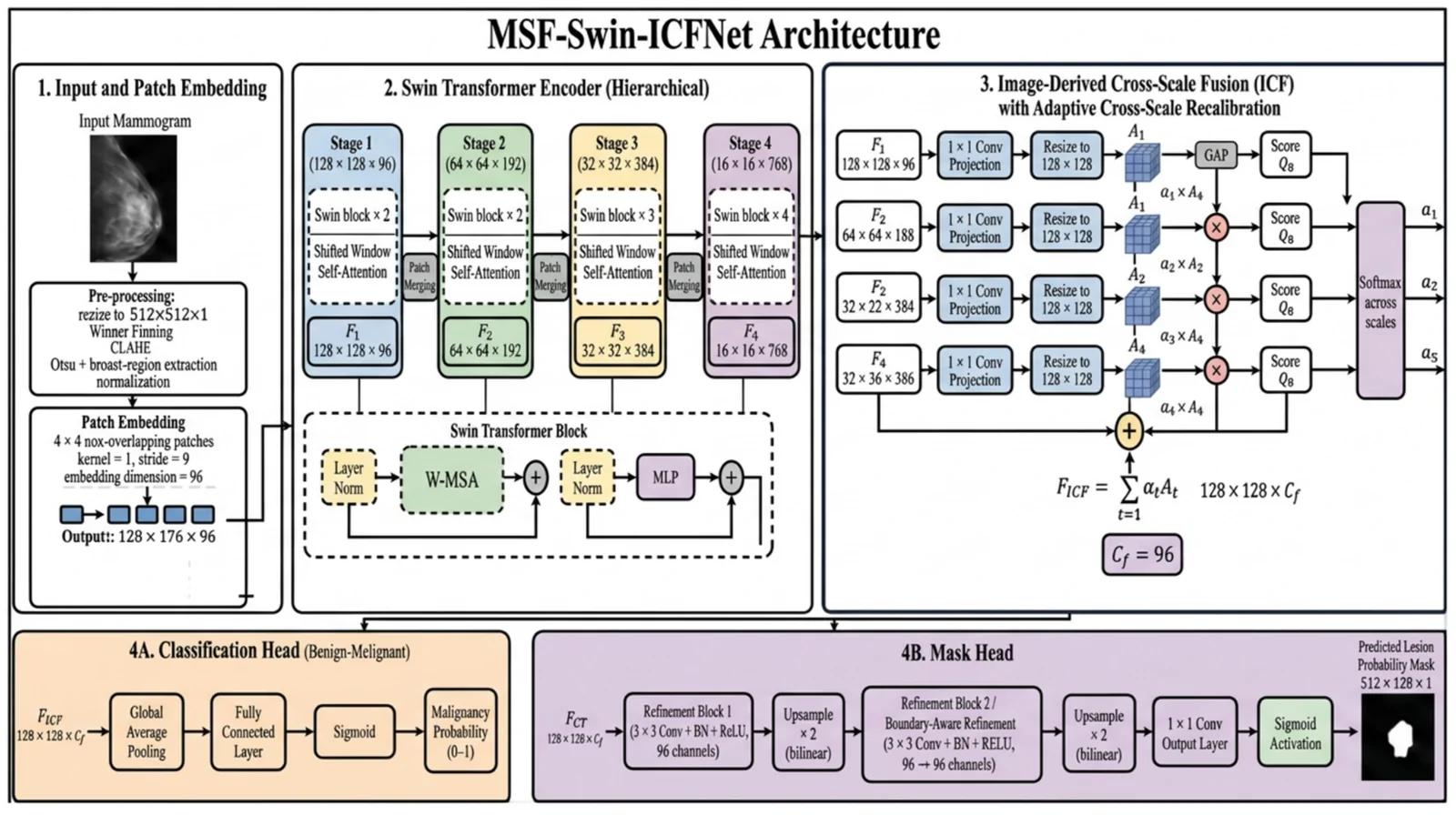
Overall architecture of MSF-Swin-ICFNet. The framework comprises mammogram preprocessing and patch embedding, four-stage hierarchical Swin Transformer feature extraction, Image-Derived Cross-Scale Fusion with adaptive feature recalibration, and shared classification and lightweight boundary-aware lesion-localization pathways. Figure notation and abbreviations: Colored boxes represent different processing stages and functional modules. Blue, green, yellow, and purple boxes denote the hierarchical Swin Transformer stages (Stages 1–4), while orange and lavender regions represent the classification and mask heads, respectively. Dashed-line boxes indicate processing blocks or grouped operations, and solid arrows indicate the direction of feature/data flow. Circular symbols denote feature addition or fusion operations. BN = Batch Normalization; ReLU = Rectified Linear Unit; GAP = Global Average Pooling; MLP = Multi-Layer Perceptron; ICF = Image-Derived Cross-Scale Fusion.

This design differs from the previously published SwinCAMF-Net framework, which integrated mammographic views, 3D ROI-derived features, and clinical metadata using a multimodal cross-attention fusion module. In contrast, MSF-Swin-ICFNet focuses only on mammographic image-derived feature fusion and is intended for settings where clinical metadata or volumetric ROI inputs are unavailable or inconsistent. Figure 1 presents the revised architecture of the proposed model.

For breast lesion classification, the proposed architecture uses mammographic image features extracted through the Swin Transformer encoder and the proposed model. The preprocessed mammogram is divided into image patches and passed through multiple Swin Transformer stages to generate hierarchical feature representations. Multi-resolution feature maps from different stages are aligned and fused to combine low-level boundary information with high-level semantic representations. The fused image-derived representation is then passed to the classification head to predict whether the lesion is benign or malignant. For lesion localization, the lightweight cross-scale mask head refines the fused features and generates a lesion mask using boundary-aware feature refinement. Unlike the previously published SwinCAMF-Net framework, the present model does not include a clinical projector, 3D volume encoder, CAF/CAFM module, or external volumetric ROI input. The proposed feature refinement operates only on mammographic image-derived feature maps.

**Figure 2:**
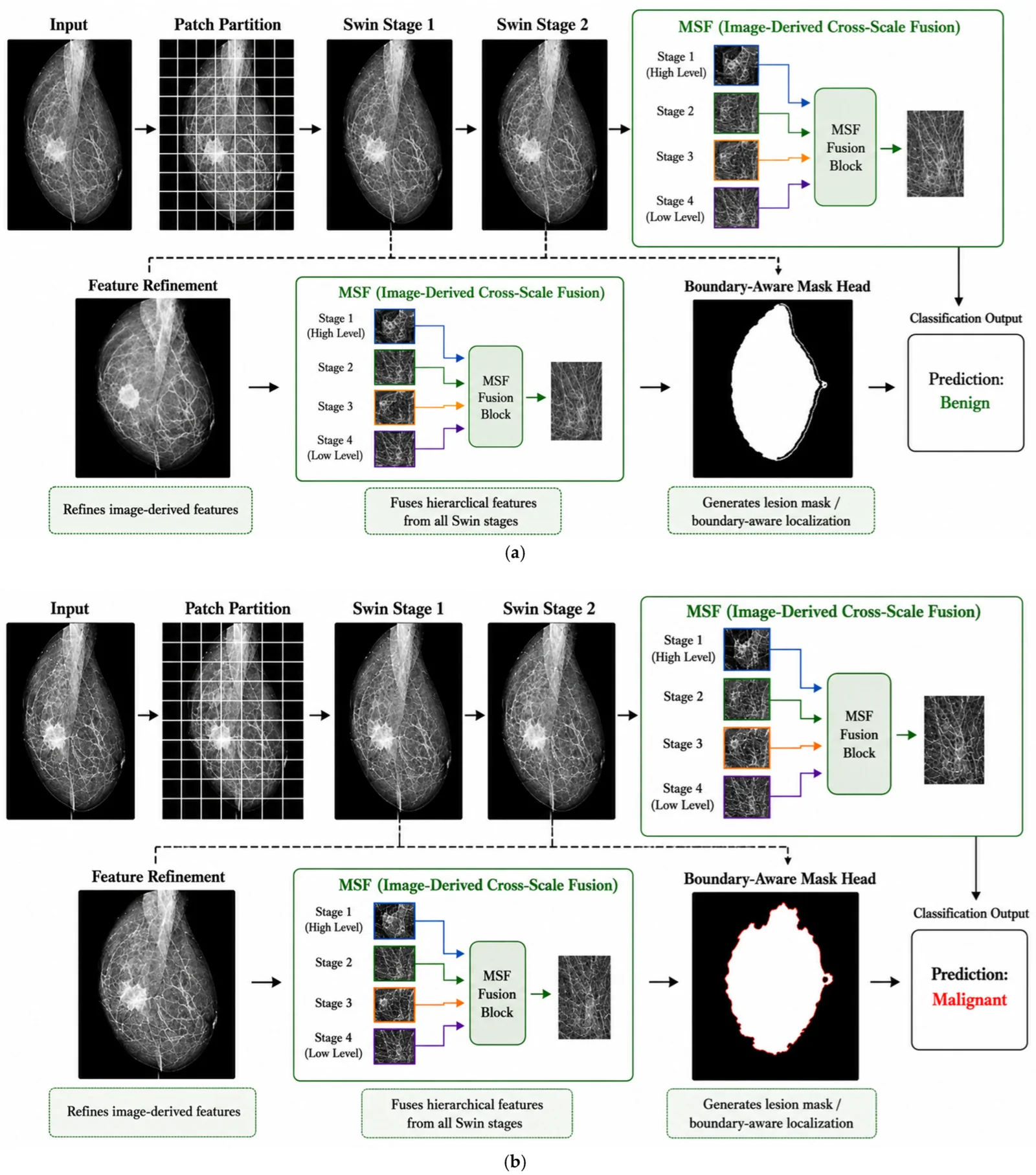
Stage-wise workflow of MSF-Swin-ICFNet for representative benign and malignant mammograms, illustrating image input, patch partitioning, hierarchical Swin feature extraction, Image-Derived Cross-Scale Fusion, feature refinement, boundary-aware lesion localization, and final classification. (**a**) Benign example; (**b**) malignant example. Figure notation: Blue, green, orange, and purple arrows/outlines represent features from Swin Transformer Stages 1, 2, 3, and 4, respectively, with Stage 1 representing the highest-resolution feature level and Stage 4 the lowest-resolution feature level. Rounded green boxes denote the Image-Derived Cross-Scale Fusion (MSF) and feature-processing modules. Solid arrows indicate the direction of image and feature flow, while dashed lines indicate feature connections from the Swin Transformer stages to subsequent processing modules. MSF = Image-Derived Cross-Scale Fusion. Black-and-white image panels represent the input mammogram and image-derived feature representations. Colored outlines and arrows indicate the corresponding hierarchical Swin Transformer stages: blue = Stage 1, green = Stage 2, yellow/orange = Stage 3, and purple = Stage 4. Rounded green boxes denote the MSF (Image-Derived Cross-Scale Fusion) and feature-processing modules. Dashed lines indicate feature connections between the Swin stages and subsequent processing modules, while solid arrows indicate the direction of image/feature flow. MSF = Image-Derived Cross-Scale Fusion.

Figure 2 illustrates the stage-wise workflow of the proposed image-derived model for benign and malignant prediction. The workflow shows patch partitioning, Swin Transformer feature extraction, image-derived cross-scale fusion, feature refinement, boundary-aware mask generation, and final classification output.

## 5. Training and Performance

The MSF-Swin-ICFNet model was trained and evaluated using CBIS-DDSM and RTM datasets. Each dataset was divided into training, validation, and testing subsets using a 70:15:15 ratio. Final performance was measured using standard metrics for classification, segmentation, and computational efficiency for both mammogram-level classification and pixel-level lesion mask prediction.

### 5.1. Training and Evaluation

The MSF-Swin-ICFNet model was trained and evaluated using CBIS-DDSM and RTM mammographic datasets. Each dataset was divided into training, validation, and testing subsets using a stratified 70:15:15 allocation with a fixed random seed. Stratification preserved the benign–malignant distribution, while grouping by patient identifier ensured that all mammograms belonging to one patient remained within a single partition. Patient–image partitioning was performed in two stages. First, all mammograms, standard views, lesion annotations, masks, and derived samples were grouped according to a unique canonical patient identifier. A single diagnostic label was then assigned at the patient level before partitioning. Patients were stratified according to benign–malignant status and allocated to training, validation, and testing subsets in a 70:15:15 ratio using a fixed random seed of 42. After the patient-level assignment was completed, every mammogram and associated annotation belonging to a patient inherited that patient’s partition label. Thus, images were not independently randomized across the three subsets, and mammograms originating from the same patient could not be distributed between training, validation, and testing sets. Data augmentation was performed only after this patient-level split and was restricted to the training subset. For segmentation, the mask-bearing mammograms retained the partition assignment of their corresponding source patient; the segmentation subset was therefore not repartitioned independently. For grouped cross-validation, patients from the development cohort were assigned to folds as intact groups, while the independent test cohort remained excluded from cross-validation. Consequently, the training, validation, and testing subsets were mutually exclusive at the patient level; a patient assigned to one subset was not permitted to appear in either of the other subsets. All mammograms, views, lesion annotations, masks, and derived samples associated with the same patient identifier inherited the same partition assignment. Patient identifiers were checked across the partitions for potential overlap before model training and evaluation. This patient-disjoint partitioning strategy was adopted to prevent patient-level information leakage between model-development and test cohorts. Detailed patient-level split and leakage-verification results are provided in Supplementary Table S2.

For CBIS-DDSM, the final patient-level cohort comprised 891 unique patients and 1592 full mammograms. The patient-level split contained 624 training patients, 133 validation patients, and 134 test patients. The training cohort comprised 301 benign and 323 malignant patients; the validation cohort comprised 65 benign and 68 malignant patients; and the test cohort comprised 65 benign and 69 malignant patients. The corresponding image counts were 1114 training mammograms, 239 validation mammograms, and 239 test mammograms. For the RTM cohort, patient-level partitioning was completed before augmentation and model training. The 573 patients were divided using a stratified 70:15:15 allocation with random seed 42, resulting in 401 training patients, 86 validation patients, and 86 test patients. These partitions comprised 7083, 1498, and 1489 mammograms, respectively. The corresponding benign–malignant patient distributions were 215/186 in the training cohort, 46/40 in the validation cohort, and 46/40 in the test cohort. No patient identifier occurred in more than one partition.

The model accepted an individual mammogram as input, and the primary classification metrics reported in the main manuscript were calculated at the mammogram level using images from patient-disjoint test cohorts. Complete patient-level partition counts grouped cross-validation details, probability-aggregation procedures, and patient-level results are reported in the Supplementary Materials. For comparative evaluation, all baseline and state-of-the-art architectures were reimplemented within the same experimental framework. Each comparator model used the identical patient-level training, validation, and testing partitions employed for MSF-Swin-ICFNet. The same image preprocessing, training-only augmentation policy, and performance metrics were applied across models. The patient-disjoint test cohorts were kept fixed throughout the comparative experiments so that the reported performance differences reflected model behaviour under a common evaluation setting rather than differences in dataset partitioning.

Several leakage-control mechanisms were incorporated into the experimental protocol. First, data partitioning was performed at the patient level before model training, so that all mammograms, views, lesion annotations, masks, lesion crops, and derived samples associated with a patient remained within a single training, validation, or testing subset. Second, data augmentation was applied only after patient-level partitioning and was restricted to the training subset; no augmented samples were introduced into the validation or test cohorts. Third, for segmentation, mask-bearing mammograms retained the partition assignment of their source patient and were not repartitioned independently. Fourth, grouped cross-validation was performed using complete patient groups, while the independent test cohort remained excluded from model development and cross-validation. Finally, model-selection decisions and operating-threshold selection were based on the validation cohort, while the test cohort was retained for final evaluation. These procedures were adopted to minimize direct and indirect information leakage between model-development and evaluation cohorts.

To ensure a controlled comparison, all reimplemented baseline and state-of-the-art models were trained and evaluated under the same experimental conditions as MSF-Swin-ICFNet. The same patient-disjoint training, validation, and testing partitions were used for every model, and no model-specific repartitioning of the datasets was performed. All models received identically preprocessed mammograms, and the same training-only augmentation policy was applied. The same batch size, computing environment, model-selection procedure, and evaluation metrics were maintained across all comparative experiments. The test cohorts remained fixed throughout the experiments and were used only for final evaluation. Architecture-specific layers and structural components were retained according to the respective original model designs; however, the data-processing and evaluation protocol was kept identical across models to provide a fair comparison.

Accordingly, the training, validation and testing subsets were mutually exclusive at the patient level; a patient represented in one subset was not permitted to appear in either of the other subsets. The partitioning procedure was designed so that a patient identifier could occur in only one of the training, validation, or testing subsets. The model accepted an individual mammogram as input, and the primary classification metrics reported in the main manuscript were calculated at the mammogram level using images from patient-disjoint test cohorts. Complete patient-level partition counts grouped cross-validation details, probability-aggregation procedures, and patient-level results are reported in the Supplementary Materials. All 10,070 eligible RTM mammograms were used for classification model development. For the segmentation task, the analytical subset was restricted to the 4863 mammograms having corresponding provider-supplied lesion masks. Each mammogram and its corresponding lesion mask inherited the same patient-level partition assignment. Mammograms without lesion masks contributed to classification training and evaluation but did not contribute to the segmentation loss or segmentation evaluation.

### 5.2. Performance Metrics

The performance of MSF-Swin-ICFNet was evaluated using classification, segmentation, and efficiency metrics.

Primary classification performance in the main manuscript was evaluated at the mammogram level using accuracy, precision, recall, specificity, F1-score, ROC-AUC, and PR-AUC. Each test mammogram was treated as one prediction; however, every test mammogram originated from a patient who was absent from the training and validation cohorts. The separate patient-level analysis, including probability aggregation and patient-level bootstrap confidence intervals, is presented in Supplementary Tables S1 and S4–S6.

For segmentation, the model was evaluated using the Dice coefficient, Intersection over Union, precision, recall, and Hausdorff distance. Dice coefficient and IoU measured the overlap between predicted lesion regions and ground-truth masks. Precision and recall assessed pixel-level segmentation reliability, while Hausdorff distance measured boundary-level localization accuracy. For computational efficiency, inference time, training time, model size, parameter count, memory usage, throughput, and FLOPs were reported to assess the practical feasibility of the proposed model.

For uncertainty estimation, two-sided 95% confidence intervals were estimated using patient-clustered bootstrap resampling with 1000 bootstrap repetitions. The patient was treated as the statistical resampling unit because multiple mammograms could originate from the same patient. In each bootstrap replicate, patients were sampled with replacement, and all mammograms belonging to a selected patient were retained together. For patient-level classification analysis, the effective resampling sample size was 134 patients for CBIS-DDSM and 86 patients for RTM. For segmentation, the effective resampling sample size was 134 unique test patients for CBIS-DDSM and 86 unique test patients for RTM. The confidence limits were obtained using the percentile method, using the 2.5th and 97.5th percentiles of the bootstrap distributions. This patient-clustered procedure accounts for within-patient correlation and avoids treating multiple mammograms from the same patient as statistically independent observations.

### 5.3. Processor and Hardware Configuration

All experiments were conducted using an NVIDIA A100 GPU with 40 GB memory and a batch size of 8. The proposed MSF-Swin-ICFNet required an average training time of 10.8 min per epoch, with a total training time of approximately 7.2 h. Peak training memory usage was 31.9 GB, and the average training speed was 25.1 samples per second. During inference, the model required approximately 0.26 s per sample, with an inference memory usage of 5.6 GB and a throughput of 13,846 samples per hour. Each inference required 42.7 GFLOPs. The final model contained 87.2 million total parameters, of which 84.6 million were trainable. The model size was 312.7 MB, and the final feature dimension was 1024. Table 8 presents the hardware and computational configuration details of the proposed model.

## 6. Visualization of Results

This section presents the quantitative and visual evaluation of MSF-Swin-ICFNet on the CBIS-DDSM and RTM mammographic datasets. The analysis includes classification performance, segmentation performance, cross-validation results, ablation analysis, attention visualization, and comparison with existing transformer-based and hybrid models. Visual explanations are used to assess whether the model focuses on clinically relevant lesion regions during prediction. The proposed method generated stable classification and segmentation results on both datasets. The Swin Transformer encoder captured hierarchical image features, while the Multi-Scale Feature Fusion module combined low-level boundary information with high-level semantic representations. The proposed framework enhanced diagnostically relevant regions, improving the model’s ability to classify and localize lesions, especially in cases with irregular shape, low contrast, dense breast tissue, or abnormal tissue patterns.

### 6.1. Computational Performance of the Model

The computational performance of MSF-Swin-ICFNet was evaluated using an NVIDIA A100 GPU with 40 GB memory and a batch size of 8. The model required an average training time of 10.8 min per epoch, with a total training time of approximately 7.2 h. Peak training memory usage was 31.9 GB, and the average training speed was 25.1 samples per second. During inference, the model required approximately 0.26 s per sample, with an inference memory usage of 5.6 GB and a throughput of 13,846 samples per hour. Each inference required 42.7 GFLOPs. The final model contained 87.2 million total parameters, of which 84.6 million were trainable. The final model size was 312.7 MB, with a feature dimension of 1024. These results indicate that MSF-Swin-ICFNet provides a practical balance between computational complexity and joint diagnostic and lesion-localization performance. Although the model requires a high-memory GPU during training, its inference time is suitable for offline mammographic image analysis and research-level decision-support applications.

### 6.2. Segmentation Performance

Segmentation performance was evaluated at the mammogram level using images with corresponding lesion masks. The proposed MSF-Swin-ICFNet achieved a Dice coefficient of 0.786 ± 0.041 on CBIS-DDSM and 0.878 ± 0.046 on RTM. The corresponding IoU values were 0.654 ± 0.053 and 0.791 ± 0.052, respectively. Pixel-level precision was 0.822 ± 0.039 for CBIS-DDSM and 0.860 ± 0.041 for RTM, while pixel-level recall was 0.819 ± 0.035 and 0.914 ± 0.043, respectively. The corresponding HD95 values were 3.24 ± 0.89 mm and 2.96 ± 0.88 mm. The two-sided 95% confidence intervals were 0.779–0.793 for CBIS-DDSM Dice and 0.870–0.886 for RTM Dice, with corresponding intervals for the remaining metrics reported in Table 9.

The segmentation results in Table 8 show a clear difference in localization behaviour between the two evaluated cohorts, with RTM exhibiting greater region overlap and recall together with somewhat better boundary agreement, whereas CBIS-DDSM represents a comparatively more challenging segmentation setting. This difference should not be attributed directly to the proposed architecture because CBIS-DDSM and RTM differ in cohort origin, acquisition environment, lesion composition, image characteristics, and annotation provenance. The supplementary analysis provides the corresponding patient-disjoint segmentation results and confidence intervals in Supplementary Table S7.

Several dataset-related mechanisms may contribute to this observation. First, differences in mammography acquisition systems, image-processing characteristics, spatial resolution, contrast, and overall image quality may alter lesion conspicuity and boundary visibility. However, sufficiently detailed acquisition information was not available for a controlled equipment-specific comparison between the two cohorts. Second, segmentation difficulty may vary according to lesion size, morphology, lesion-to-background contrast, and breast composition. Third, reference-annotation characteristics can substantially influence overlap- and boundary-based segmentation measures. The RTM masks were supplied by the dataset provider, but information concerning the original delineation procedure, number and expertise of annotators, inter-observer variability, and consensus process was unavailable. Consequently, differences in annotation consistency cannot be excluded as a contributor to the observed performance gap.

Consequently, the higher RTM segmentation scores should not be interpreted as evidence that the model is intrinsically more effective on clinically acquired mammograms. A dedicated cross-dataset analysis would be required to determine the origin of the observed performance difference. Such an analysis should stratify segmentation performance according to lesion size, morphology, contrast, breast density, and image-quality characteristics and should, where possible, examine annotation variability and boundary agreement. These analyses would help determine whether the difference between CBIS-DDSM and RTM arises primarily from lesion characteristics, acquisition quality, reference-mask properties, or interactions among these factors. Therefore, the present cross-dataset difference is reported as an empirical observation rather than a causal finding.

**Figure 3:**
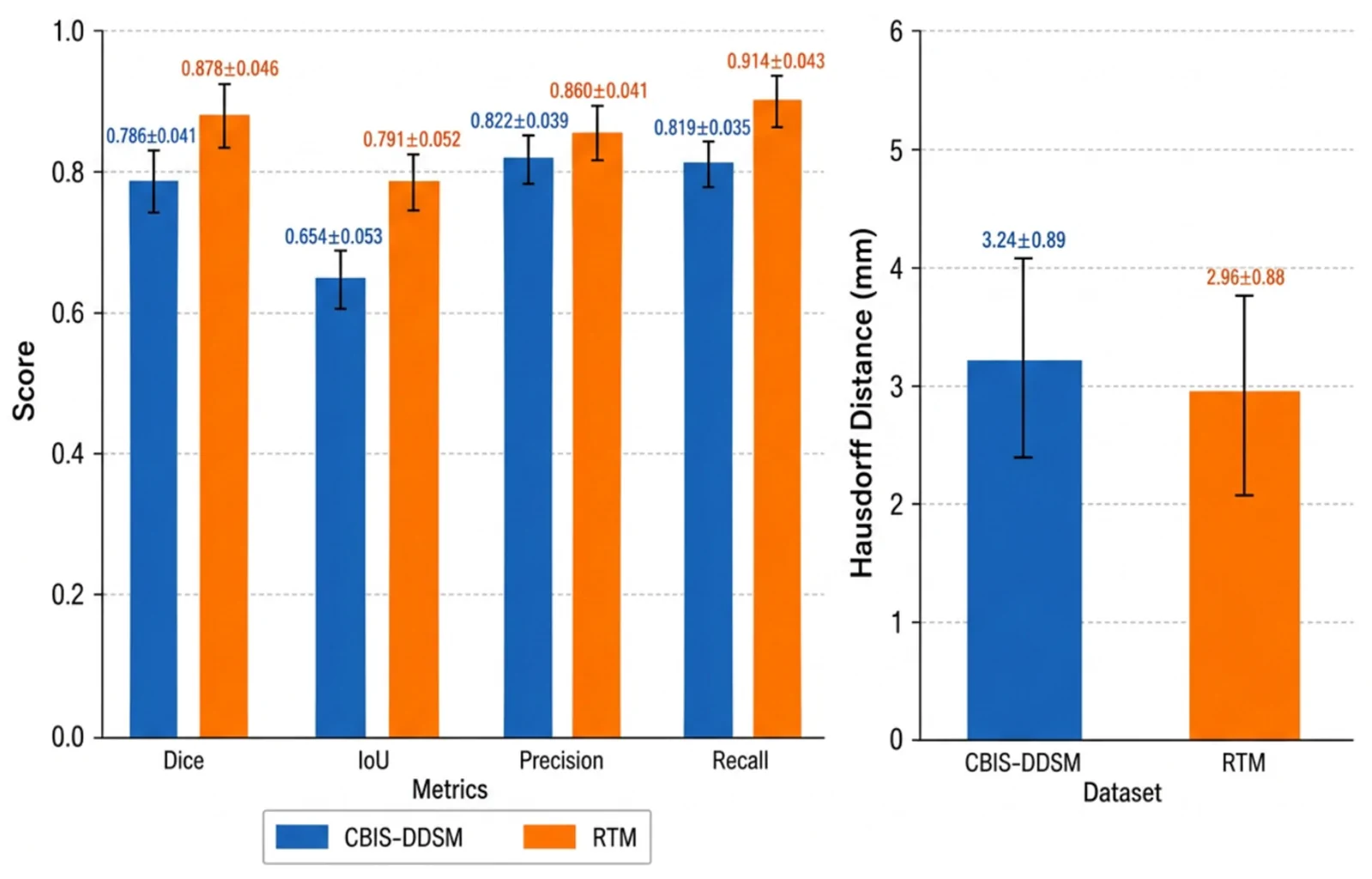
Segmentation performance comparison of MSF-Swin-ICFNet on CBIS-DDSM and RTM datasets.

Figure 3 compares the Dice coefficient, IoU, precision, recall, and Hausdorff distance across both datasets. Higher Dice, IoU, precision, and recall values indicate better segmentation quality, while lower Hausdorff distance indicates improved boundary accuracy.

### 6.3. Mammogram-Level Classification Performance

The classification performance reported in this section was calculated at the individual-mammogram level. Although each mammogram was processed and scored independently, the test images were obtained from patient-disjoint cohorts; therefore, no patient contributing a test mammogram was represented in the training or validation subsets. Patient-level probability aggregation, patient-level classification metrics, and patient-level confusion matrices are reported separately in the Supplementary Materials. Patient-level ROC-AUC results and their confidence intervals are provided in Supplementary Table S8. The results in Table 10 indicate that MSF-Swin-ICFNet maintained a favorable balance between malignant-case sensitivity and benign-case specificity within the evaluated patient-disjoint cohorts. Nevertheless, differences between CBIS-DDSM and RTM should not be interpreted as evidence that one cohort is intrinsically easier or that the model generalizes more effectively to clinical data. Apparent classification performance can be affected by cohort composition, lesion prevalence, lesion conspicuity, breast composition, acquisition environment, and other dataset-specific characteristics. Therefore, the cross-dataset results are interpreted as descriptive evidence of performance within the evaluated cohorts rather than as proof of domain-independent clinical robustness. Complementary patient-level results obtained by aggregating mammogram predictions are provided in the Supplementary Materials.

On the CBIS-DDSM patient-disjoint test cohort, MSF-Swin-ICFNet achieved a mammogram-level accuracy of 0.949, precision of 0.944, recall of 0.919, specificity of 0.971, F1-score of 0.931, ROC-AUC of 0.985, and PR-AUC of 0.949. On the RTM patient-disjoint test cohort, MSF-Swin-ICFNet achieved a mammogram-level accuracy of 0.968, precision of 0.953, recall of 0.959, specificity of 0.974, F1-score of 0.956, ROC-AUC of 0.992, and PR-AUC of 0.970. These results were calculated from 239 CBIS-DDSM mammograms and 1489 RTM mammograms belonging to patients who were not represented in the training or validation partitions. The complete mammogram-level metric set is also provided in Supplementary Table S5. Patient-level results obtained by aggregating mammogram probabilities are reported separately in Supplementary Table S4, and the corresponding performance comparison is illustrated in Supplementary Figure S1. Thus, the main manuscript results represent mammogram-level performance, whereas the supplementary analysis provides complementary patient-level evaluation. A supplementary visual comparison of mammogram-level classification performance, including ROC and precision–recall analyses, is provided in Supplementary Figure S2. The corresponding numerical patient-level confusion matrices are provided in Supplementary Table S6. Visual representations of these patient-level confusion matrices are provided in Supplementary Figure S3. Complementary patient-level classification results obtained by aggregating mammogram-level probabilities are reported in Supplementary Table S4, and the corresponding performance comparison is illustrated in Supplementary Figure S1. The complete mammogram-level metric set is also provided in Supplementary Table S5, with a visual comparison presented in Supplementary Figure S2. The corresponding numerical patient-level confusion matrices are provided in Supplementary Table S6, with their visual representations shown in Supplementary Figure S3.

**Figure 4:**
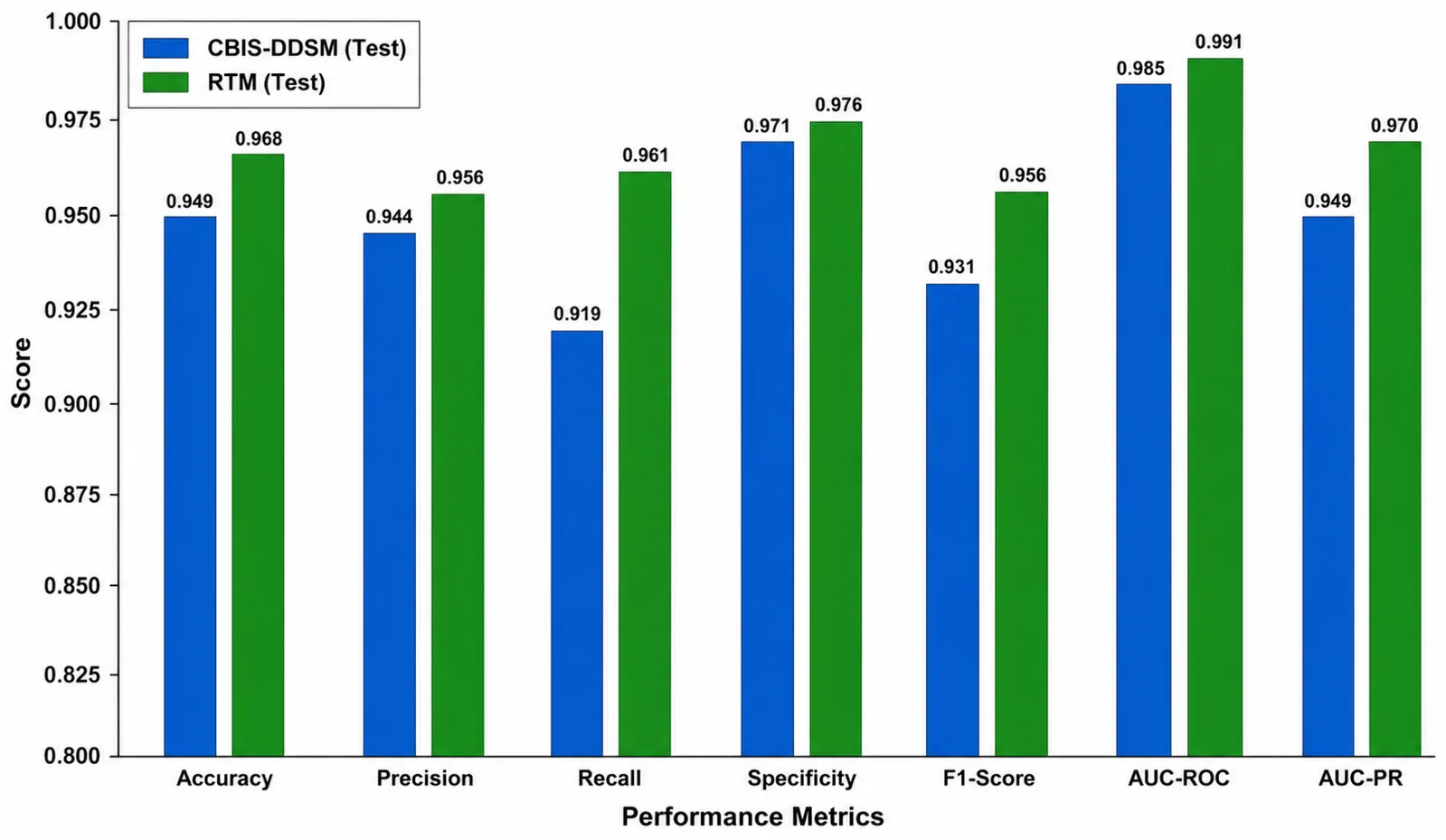
Classification performance comparison of MSF-Swin-ICFNet on CBIS-DDSM and RTM datasets.

Figure 4 presents a visual comparison of the classification metrics, including accuracy, precision, recall, F1-score, AUC-ROC, and AUC-PR, across CBIS-DDSM five-fold cross-validation, CBIS-DDSM test, and RTM test settings. The figure highlights the consistent and improved performance of the proposed model across different evaluation settings. Patient-level ROC-AUC results and their confidence intervals are provided in Supplementary Table S8, with the corresponding ROC curves shown in Supplementary Figure S4. Patient-level PR-AUC values are reported in Supplementary Tables S8 and S9 and the corresponding precision–recall curves are shown in Supplementary Figure S5.

### 6.4. F1-Score Comparison Between CBIS-DDSM and RTM

The F1-score comparison of the proposed MSF-Swin-ICFNet model with existing deep learning models is presented in Table 11. The F1-score was selected because it provides a balanced evaluation of precision and recall, which is particularly important in breast cancer classification where both false positives and false negatives have clinical significance.

The F1-score comparison in Table 10 shows that MSF-Swin-ICFNet achieved the highest F1-score among the models included in the present comparison. The previously published SwinCAMF-Net reported F1-scores of 0.902 on CBIS-DDSM and 0.944 on RTM, whereas MSF-Swin-ICFNet achieved 0.931 and 0.958, respectively. However, the SwinCAMF-Net results were taken from the previous publication and were not re-evaluated using the patient-disjoint protocol adopted in the present study. Therefore, these values are presented as contextual comparisons rather than as results from a controlled head-to-head evaluation.

**Figure 5:**
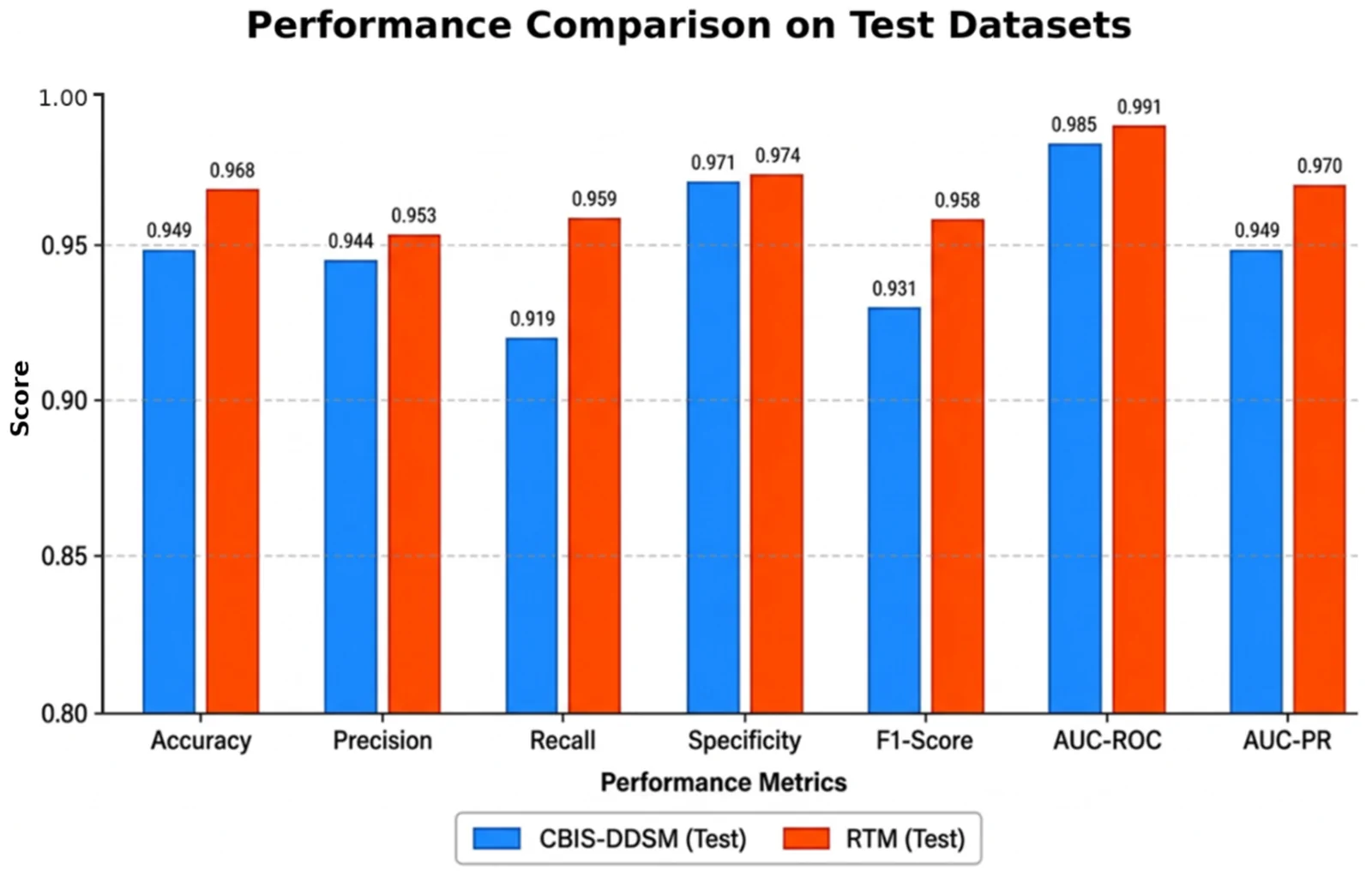
F1-score comparison of deep learning models on CBIS-DDSM and RTM datasets.

The above Figure 5 presents the F1-score performance of ResNet, DenseNet, Vision Transformer, Medical Transformer, Swin-T v2, and the proposed MSF-Swin-ICFNet model.

### 6.5. Mammogram-Level Performance Under Patient-Grouped Cross-Validation

Five-fold patient-grouped cross-validation was conducted within the RTM development cohort comprising 487 patients. The independent 86-patient test cohort remained locked and was excluded from cross-validation. All mammograms associated with a patient remained within the same fold. Approximately 97–98 patients served as validation patients in each fold, while approximately 389–390 patients were available for training in the corresponding folds. Consequently, the model was evaluated on individual images without permitting mammograms from the same patient to appear in both the training and validation portions of a fold. The complete patient and fold composition used for the five-fold patient-grouped cross-validation is provided in Supplementary Table S3. Five-fold cross-validation was conducted on the CBIS-DDSM dataset to evaluate the stability of the proposed MSF-Swin-ICFNet model across different data partitions. Unlike a single train–test split, cross-validation provides a more reliable estimate of model generalization by evaluating performance across multiple folds.

The CBIS-DDSM five-fold cross-validation results showed an accuracy of 0.881 ± 0.012, precision of 0.864 ± 0.019, recall of 0.867 ± 0.018, and F1-score of 0.858 ± 0.012. The corresponding AUC-ROC and AUC-PR values were 0.931 ± 0.015 and 0.907 ± 0.011, respectively. These results indicate stable discrimination between benign and malignant cases across different validation folds. Five-fold patient-grouped cross-validation was conducted on the CBIS-DDSM development cohort to assess the stability of the proposed model across different patient partitions. The RTM dataset was evaluated using its own patient-disjoint training, validation, and held-out test subsets and therefore represents an additional clinical-cohort evaluation rather than a fully external validation cohort. The patient-level confusion matrices were verified against the corresponding test-cohort composition. For CBIS-DDSM, the four cells sum to 134 patients (62 TN + 3 FP + 6 FN + 63 TP), comprising 65 benign and 69 malignant patients. For RTM, the four cells sum to 86 patients (44 TN + 2 FP + 2 FN + 38 TP), comprising 46 benign and 40 malignant patients. Thus, the confusion-matrix counts are consistent with the stated patient-level test cohorts. Accordingly, RTM performance should be interpreted as evidence of model performance on a clinically acquired dataset with different image characteristics, but not as independent external clinical validation and is therefore reported separately in Table 12.

**Figure 6:**
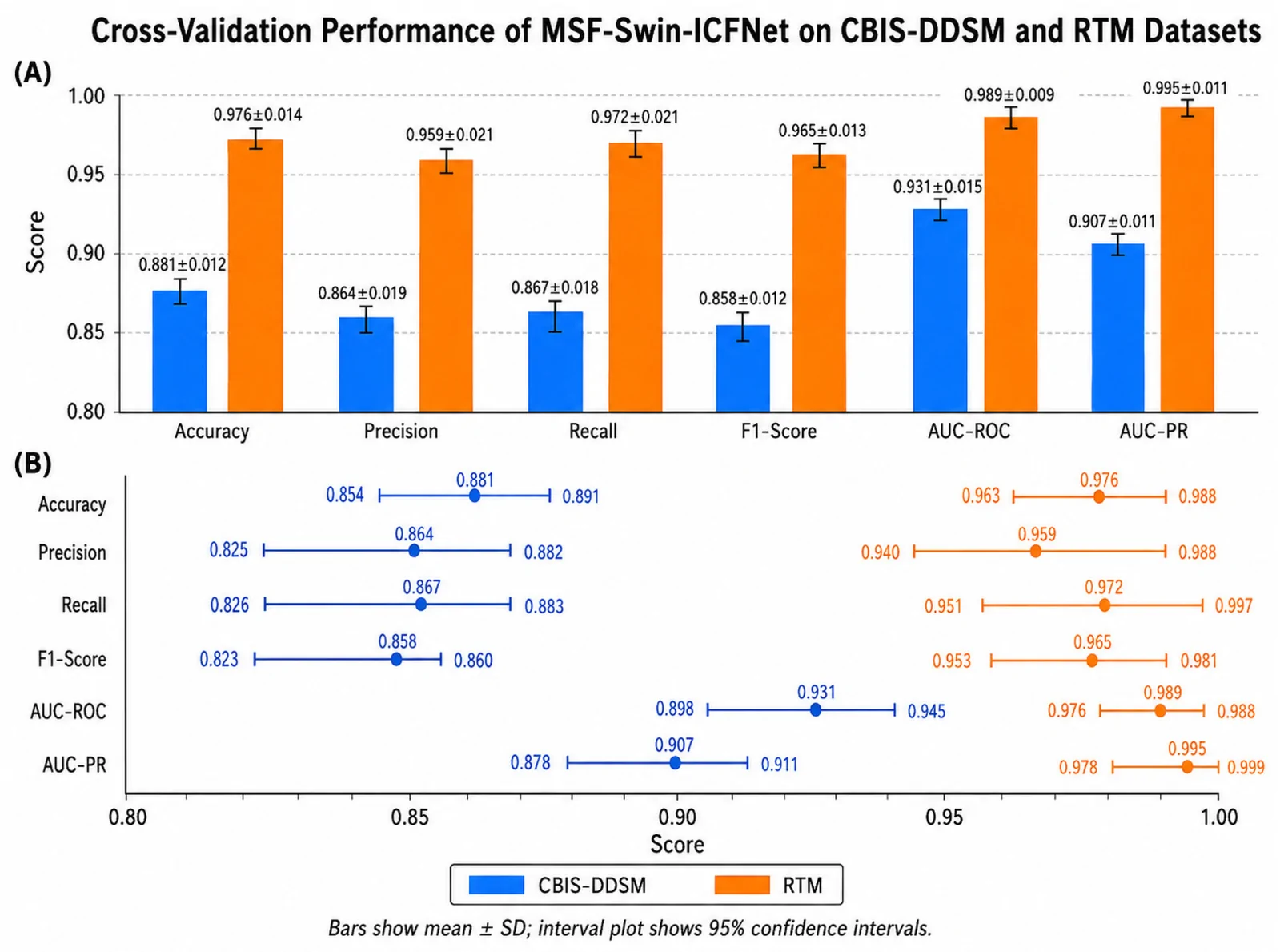
Cross-validation performance comparison of MSF-Swin-ICFNet on the CBIS-DDSM and RTM datasets. (**A**) Performance scores with standard deviations; (**B**) Performance scores with 95% confidence intervals across the evaluated metrics. (**B**): The blue circles represent the mean cross-validation scores of MSF-Swin-ICFNet on the CBIS-DDSM dataset, with the blue horizontal lines indicating the corresponding 95% confidence intervals. The orange circles represent the mean scores on the RTM dataset, with the orange horizontal lines indicating the corresponding 95% confidence intervals. The plot presents the confidence intervals for Accuracy, Precision, Recall, F1-Score, AUC-ROC, and AUC-PR across the five-fold cross-validation results.

Figure 6 presents the mean cross-validation scores for accuracy, precision, recall, F1-score, AUC-ROC, and AUC-PR. The results show that the RTM dataset achieved higher values across all major performance metrics, while the CBIS-DDSM results remained stable with narrow confidence intervals. This supports the robustness of the proposed model across different mammographic datasets.

**Figure 7:**
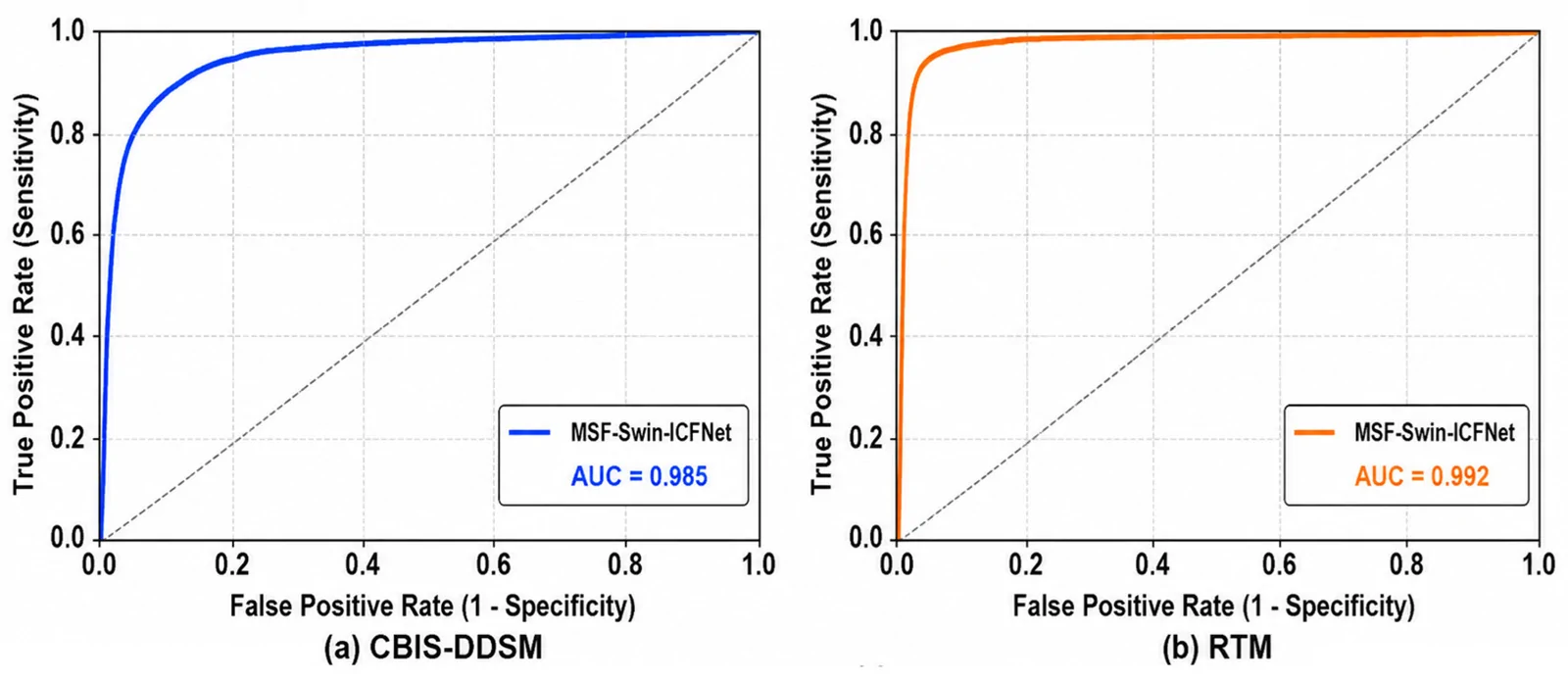
Mammogram-level ROC curves of MSF-Swin-ICFNet on patient-disjoint CBIS-DDSM and RTM test cohorts. Color and line notation: In subfigure (**a**), the blue solid line represents the ROC curve of MSF-Swin-ICFNet on the CBIS-DDSM test dataset (AUC = 0.985). In subfigure (**b**), the orange solid line represents the ROC curve of MSF-Swin-ICFNet on the RTM test dataset (AUC = 0.992). In both subfigures, the dashed diagonal line represents the performance of a random classifier (AUC = 0.5).

Figure 7 shows the discrimination ability of the proposed MSF-Swin-ICFNet model on the independent patient-disjoint test cohorts, with mammogram-level ROC-AUC values of 0.985 for (a) CBIS-DDSM and 0.992 for (b) RTM.

**Figure 8:**
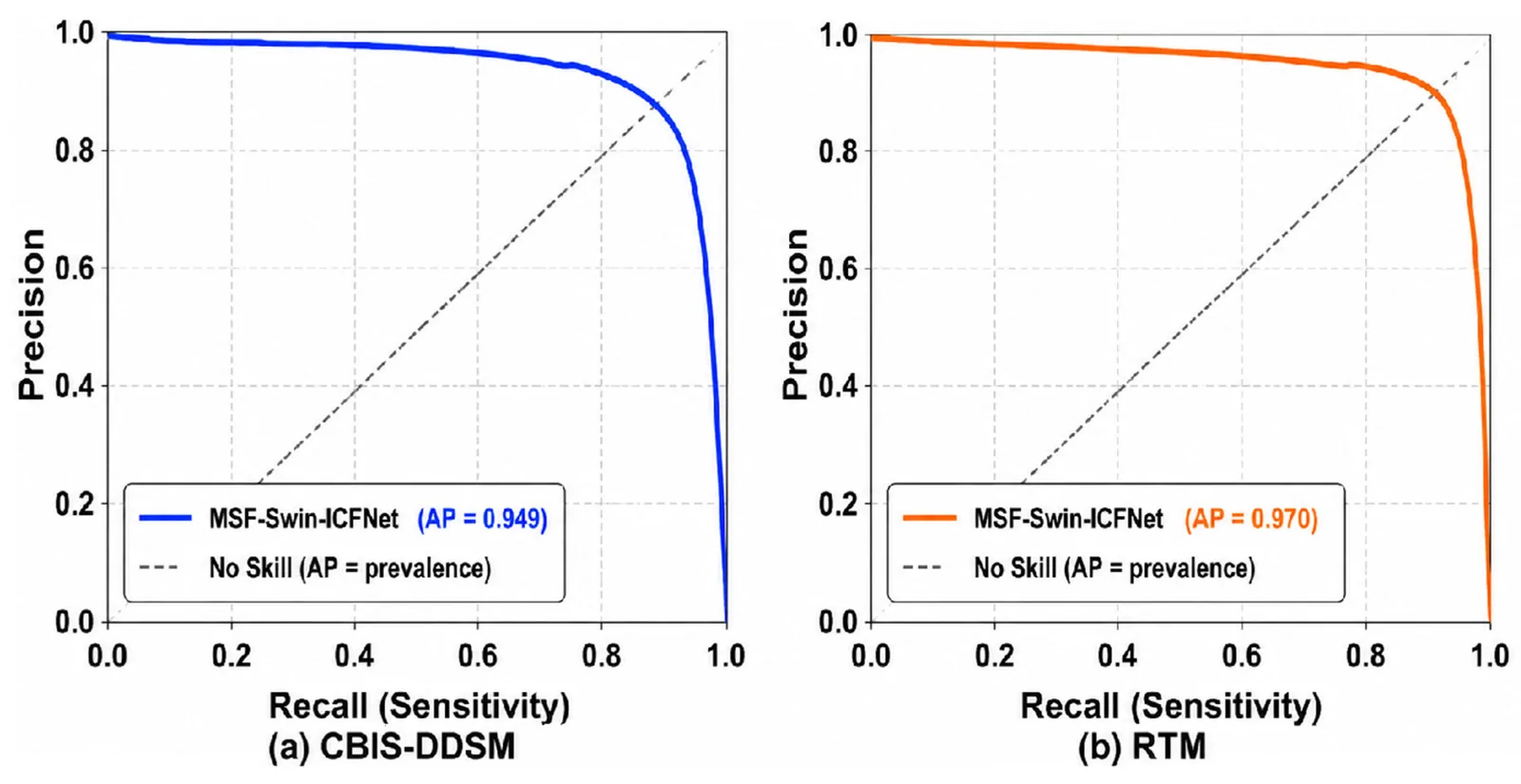
Mammogram-level precision–recall curves of MSF-Swin-ICFNet on patient-disjoint CBIS-DDSM and RTM test cohorts. Note: Corresponding patient-level ROC and precision–recall analyses are provided in Supplementary Figures S4 and S5. Note: Corresponding patient-level ROC and precision–recall analyses are provided in Supplementary Figures S4 and S5, respectively. Precision–Recall (PR) curves of MSF-Swin-ICFNet on (**a**) CBIS-DDSM and (**b**) RTM test datasets. The blue solid line in (**a**) represents the PR curve for CBIS-DDSM (AP = 0.949), while the orange solid line in (**b**) represents the PR curve for RTM (AP = 0.970). The gray dashed line represents the no-skill classifier, for which the precision corresponds to the prevalence of the positive class in the respective test dataset.

Figure 8 shows the mammogram-level precision–recall performance of the proposed MSF-Swin-ICFNet model on the independent patient-disjoint test cohorts. The model achieved PR-AUC values of 0.949 for CBIS-DDSM and 0.970 for RTM.

**Figure 9:**
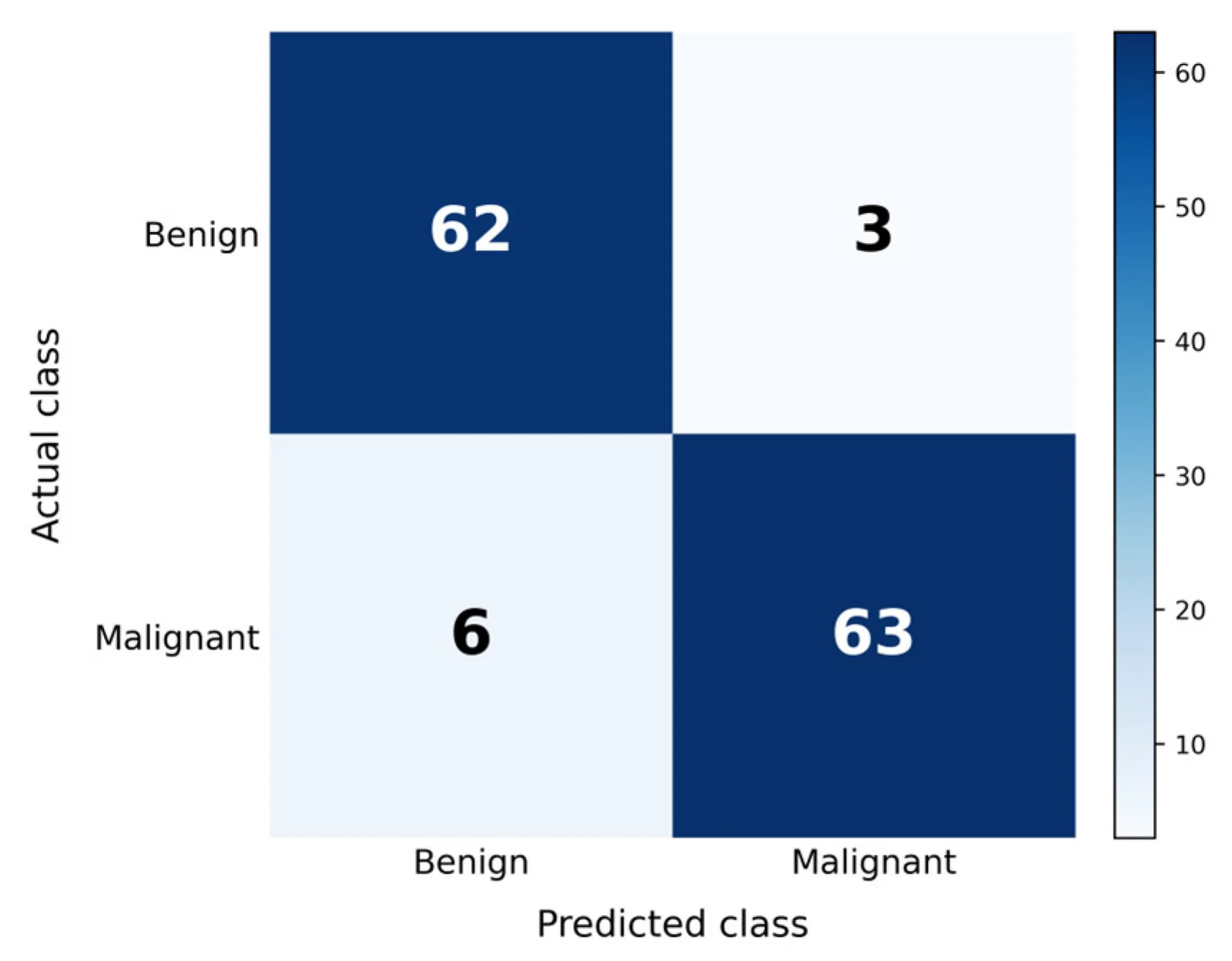
Confusion Matrix of MSF-Swin-ICFNet on the CBIS-DDSM Test Dataset.

**Figure 10:**
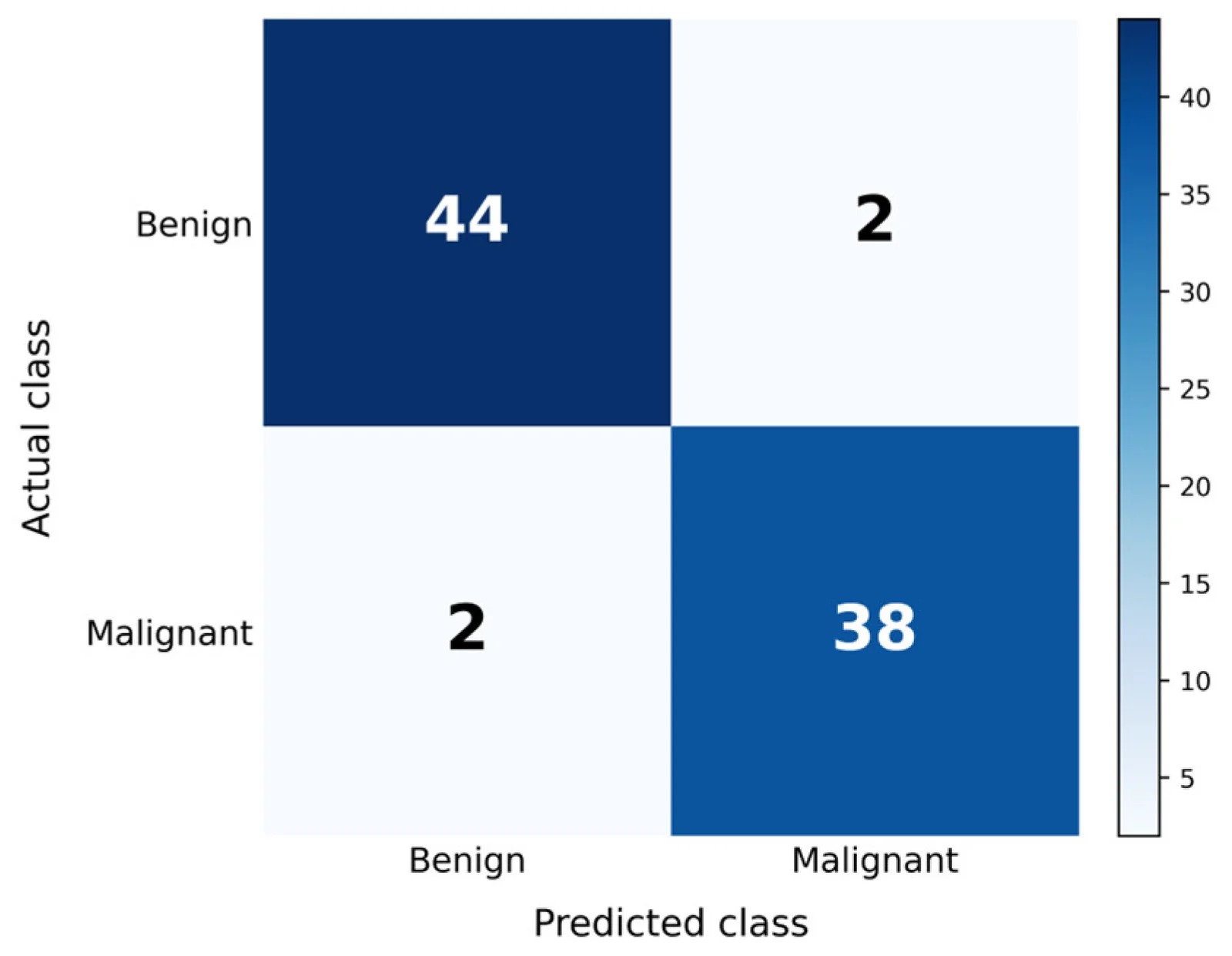
Confusion Matrix of MSF-Swin-ICFNet on the RTM Test Dataset.

Figure 9 and Figure 10 show the patient-level confusion matrices of MSF-Swin-ICFNet on the independent patient-disjoint test cohorts. For CBIS-DDSM, the test cohort contained 134 patients, comprising 65 benign and 69 malignant patients. The resulting confusion matrix contained 62 true negatives, 3 false positives, 6 false negatives, and 63 true positives. For RTM, the test cohort contained 86 patients, comprising 46 benign and 40 malignant patients, with 44 true negatives, 2 false positives, 2 false negatives, and 38 true positives. These confusion matrices correspond to the patient-level classification analysis reported in the Supplementary Materials. The corresponding patient-level PR-AUC values are reported in Supplementary Table S9. The corresponding patient-level precision–recall curves are shown in Supplementary Figure S5.

### 6.6. Ablation Study

An ablation study was conducted to evaluate the contribution of each major component in the proposed MSF-Swin-ICFNet framework. The full model was compared with reduced variants by removing the proposed module, cross-scale recalibration, feature refinement layers, and the boundary-aware mask head. The purpose of this experiment was to verify whether each image-derived component contributes meaningfully to classification and lesion mask prediction performance.

The ablation study in Table 13 demonstrates that each major component of MSF-Swin-ICFNet contributes to the overall classification and lesion-localization performance. Removing Image-Derived Cross-Scale Fusion reduced the CBIS-DDSM accuracy, F1-score, AUC-ROC, and Dice by 4.3%, 6.3%, 4.0%, and 5.6%, respectively, while the corresponding reductions for RTM were 5.6%, 6.1%, 2.0%, and 3.6%. Removing cross-scale recalibration resulted in further reductions, particularly in F1-score and Dice, indicating that adaptive weighting of the aligned feature representations contributes to the fused representation. The removal of feature-stack refinement produced larger reductions in both classification and segmentation performance, while removing the boundary-aware mask head primarily affected lesion-localization performance. The Swin Encoder Only configuration produced the lowest overall performance across both datasets, providing an additional comparison against using the backbone without the proposed fusion and refinement components. Overall, the ablation results support the contribution of the individual architectural components to the joint classification and lesion-localization framework.

**Figure 11:**
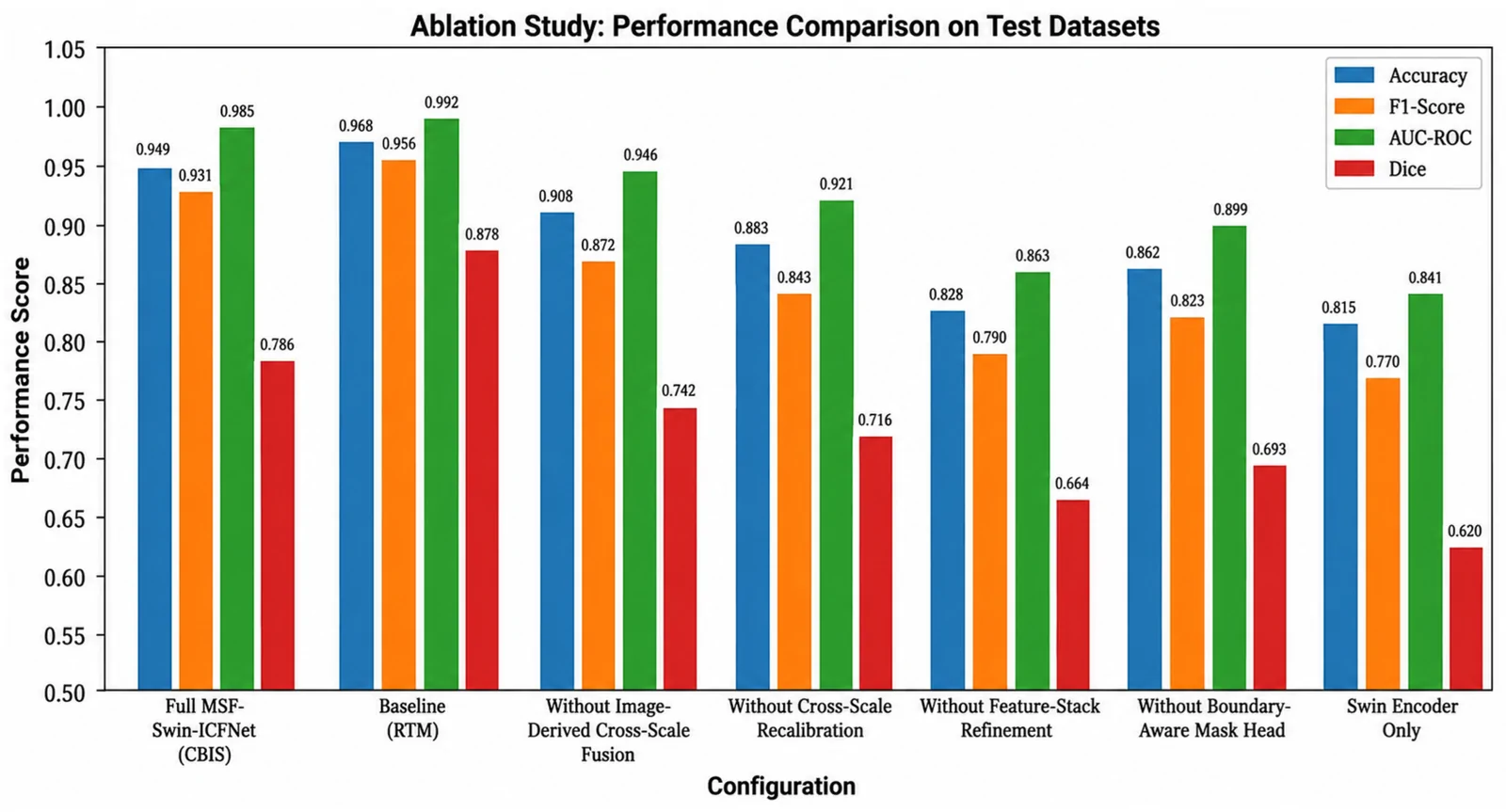
Ablation results of MSF-Swin-ICFNet on CBIS-DDSM. Performance is compared for the full model and configurations obtained by removing Image-Derived Cross-Scale Fusion (ICF), cross-scale recalibration, feature refinement, and the boundary-aware mask head, together with the Swin-encoder-only baseline.

**Figure 12:**
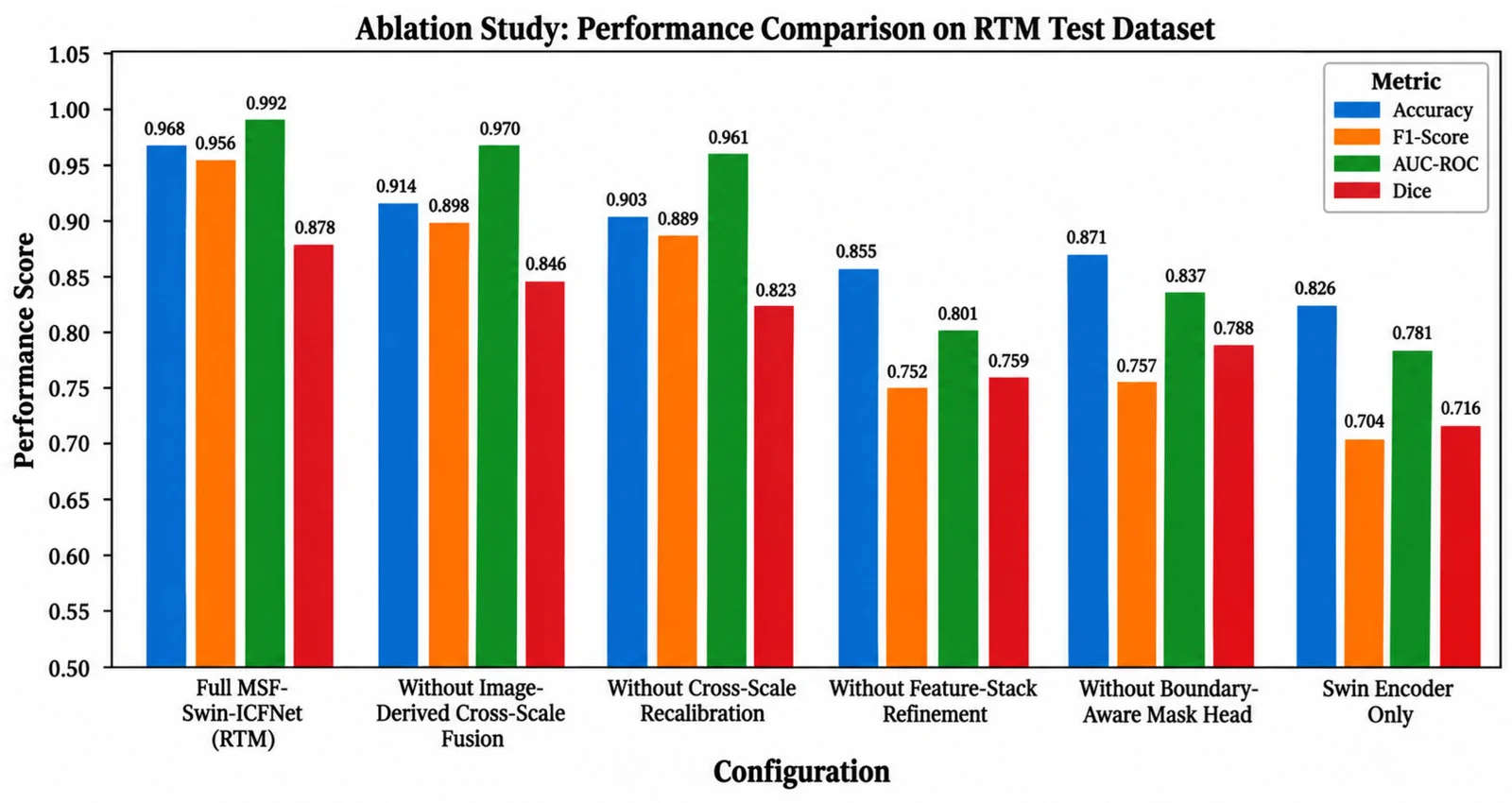
Ablation study results of MSF-Swin-ICFNet on the RTM dataset. The graph shows the effect of removing Image-Derived Cross-Scale Fusion, cross-scale recalibration, feature refinement layers, and the boundary-aware mask head from the full model.

**Figure 13:**
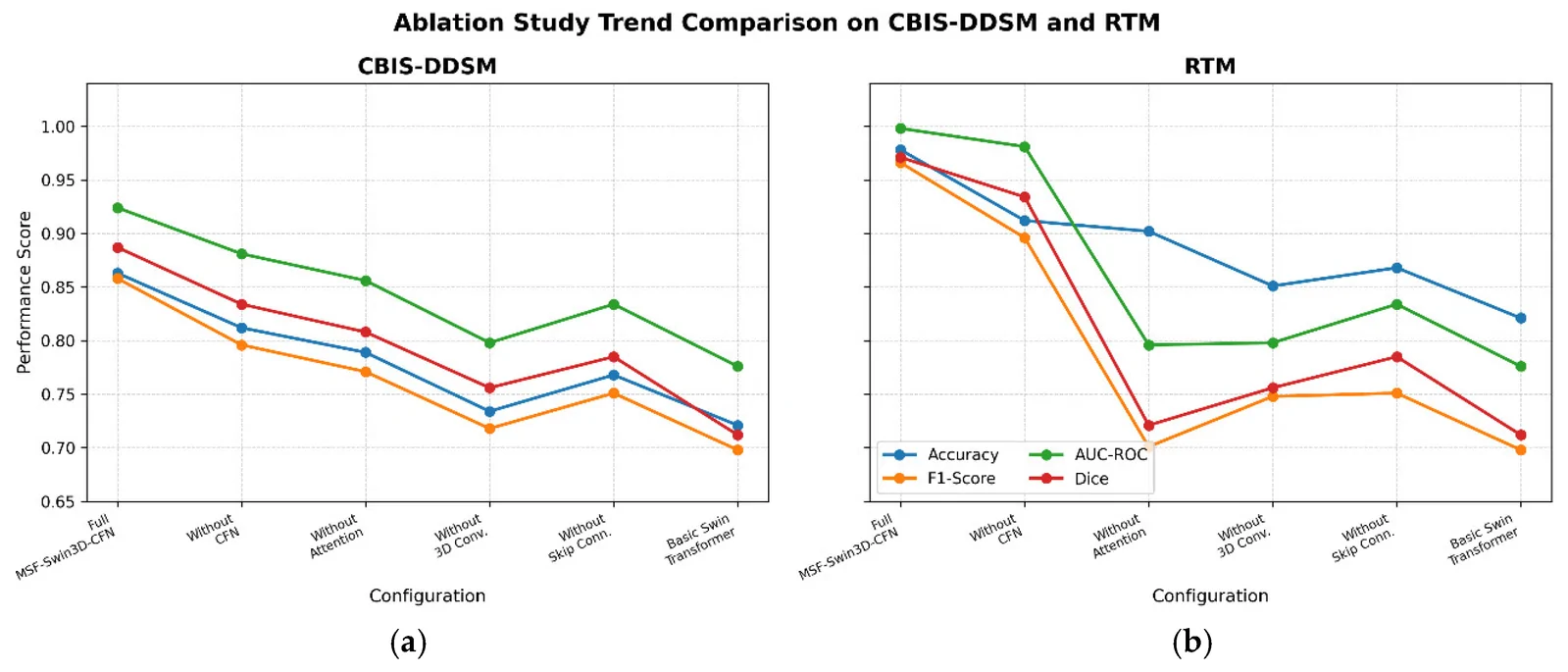
Ablation study trend comparison of MSF-Swin-ICFNet on the CBIS-DDSM and RTM test datasets. Subfigure (**a**) presents the performance trends on CBIS-DDSM, while subfigure (**b**) presents the corresponding results on RTM. In both subfigures, the blue, orange, green, and red lines represent Accuracy, F1-Score, AUC-ROC, and Dice score, respectively. The trends illustrate the changes in classification and segmentation performance when individual components of the proposed framework are removed. Color notation: In subfigure (**a**), the blue line represents Accuracy, the orange line represents F1-Score, the green line represents AUC-ROC, and the red line represents Dice score across the evaluated configurations.

Figure 11 presents the ablation results for the CBIS-DDSM dataset. The full MSF-Swin-ICFNet model shows better accuracy, F1-score, AUC-ROC, and Dice coefficient than the reduced variants, indicating that each module contributes to classification and segmentation performance. Figure 12 shows the ablation results on the RTM dataset. Removing Image-Derived Cross-Scale Fusion (ICF), cross-scale recalibration, feature refinement layers, or boundary-aware mask head reduces model performance, which confirms the importance of these components in improving feature fusion, lesion representation, and boundary localization. Figure 13 compares the ablation trends across CBIS-DDSM and RTM datasets. The trend shows that the complete model performs consistently better than all reduced configurations, while the basic Swin Transformer gives the lowest overall performance.

### 6.7. Comparison with Previous SwinCAMF-Net Study

The previously published SwinCAMF-Net and the proposed MSF-Swin-ICFNet are both based on Swin Transformer features but differ in their input modalities and fusion strategies [23]. SwinCAMF-Net integrates multi-view mammograms, 3D ROI volumes, and clinical metadata through a cross-attention fusion module, followed by classification and segmentation branches. In contrast, MSF-Swin-ICFNet focuses on hierarchical image features and cross-scale feature integration without requiring additional volumetric and clinical input streams.

The previously published SwinCAMF-Net achieved F1-scores of 0.902 on CBIS-DDSM and 0.944 on RTM, while MSF-Swin-ICFNet achieved 0.931 and 0.958, respectively. For the Dice/AUC comparison, SwinCAMF-Net reported 0.795/0.957 on CBIS-DDSM and 0.931/0.977 on RTM, whereas MSF-Swin-ICFNet achieved 0.786/0.985 and 0.878/0.992, respectively. Thus, MSF-Swin-ICFNet provides higher reported classification AUC values, whereas SwinCAMF-Net provides higher reported segmentation Dice values, particularly on the RTM dataset.

SwinCAMF-Net has the advantage of incorporating complementary volumetric and clinical information, which can provide additional contextual information for lesion analysis. Its multimodal design, however, requires additional input modalities and associated processing. MSF-Swin-ICFNet uses image-derived features and therefore provides a simpler input configuration while emphasizing hierarchical cross-scale feature integration. Nevertheless, the lower Dice values obtained by MSF-Swin-ICFNet indicate that its lesion-localization performance remains below that reported for SwinCAMF-Net. Therefore, the two approaches represent different architectural trade-offs rather than one model being universally superior to the other.

Since the SwinCAMF-Net results were reproduced from the previous publication and were not re-evaluated using the exact patient-disjoint data partitions and experimental protocol of the present study, this comparison should be interpreted as contextual rather than as a definitive head-to-head evaluation.

### 6.8. Visualization of Model Performance

The visualizations of the proposed MSF-Swin-ICFNet model enable a detailed understanding of the regions of interest that relate to lesions in the process of breast cancer classification and segmentation, based on how the network focuses on the relevant areas of the image. Grad-CAM maps were generated to identify image regions that contributed most strongly to the model predictions. Correct benign and malignant predictions were examined together with false-positive and false-negative cases to assess whether the model attended to clinically meaningful lesion regions.

In TP+ classifications, the attention maps demonstrate a strong and focal activation over the regions of the image that correspond to the suspicious lesion(s), indicating that the model correctly focused on diagnostically higher abnormal areas of the image. In TN- classifications, the level of activation occurs in a less focused and distributed fashion across the imaged normal tissue representing that the model did not detect malignant patterns. In FP+ classifications, the attention areas will activate near benign structures which may visually appear similar to malignant abnormal findings such as dense tissue or atypical contour. In FN- classifications, activation of attention occurs in a weak or scattered manner and does not adequately demonstrate focus of attention on the actual lesion region.

**Figure 14:**
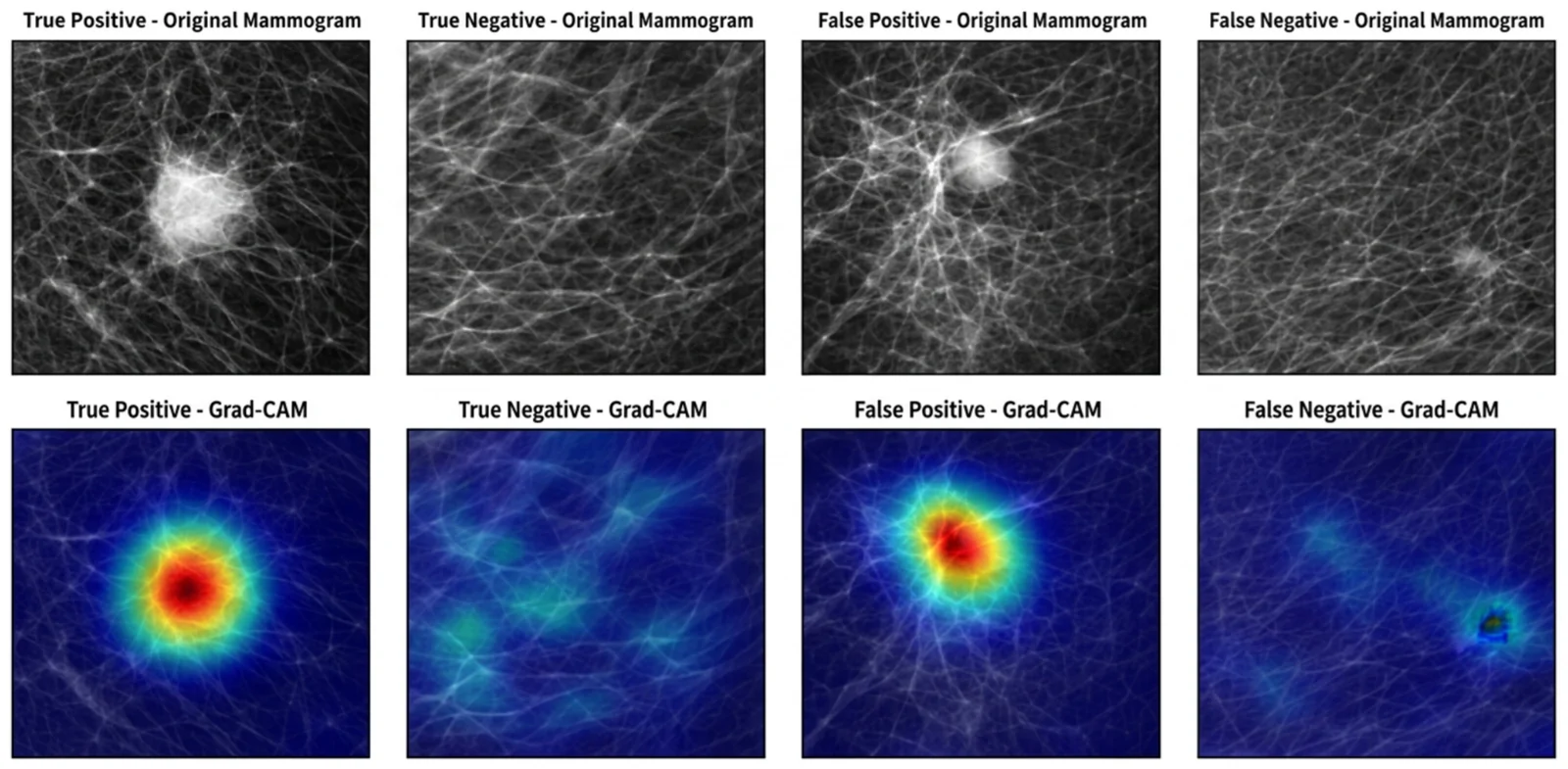
Grad-CAM visualization of MSF-Swin-ICFNet predictions on representative mammograms. The first row shows the original mammograms for true-positive, true-negative, false-positive, and false-negative cases, while the second row shows the corresponding Grad-CAM visualizations. Color interpretation: In the Grad-CAM heatmaps, blue regions indicate relatively low model attention, green and yellow regions indicate progressively higher attention, and red regions indicate the highest model attention toward the prediction. The original mammograms are displayed in grayscale. Grad-CAM: Gradient-weighted Class Activation Mapping. The heatmap visualizes the image regions contributing to the model prediction, with blue indicating lower activation and red indicating higher activation.

Overall, the Grad-CAM visualizations in Figure 14 indicate that MSF-Swin-ICFNet focuses on clinically important areas in mammograms. Therefore, these findings lend support to the interpretability of this model, while providing information about successful and unsuccessful predictions. Furthermore, these visualizations are useful for interpretability in the model, identify its limitations, and direct the improvement of its architectural features. The results of this visual analysis are consistent with the CBIS-DDSM and RTM datasets, which report the model’s performance with respect to classification and segmentation. Figure 14 shows original mammograms and their corresponding attention heatmaps for true positive, true negative, false positive, and false negative predictions. Strong localized activations indicate that the model had focused its activation on the lesion correctly, while weak or scattered activations signal the possibility of failure to correctly predict lesions.

### 6.9. Comparison with State-of-the-Art Transformer Models

To provide a controlled comparison with representative deep-learning, transformer-based, and hybrid architectures, the comparator models reimplemented in the present study were evaluated under the same experimental protocol used for MSF-Swin-ICFNet. The previously published SwinCAMF-Net results are included separately as a contextual reference and were not re-evaluated under the present protocol. Specifically, ResNet, DenseNet, Vision Transformer, Medical Transformer, Swin-T v2, ViT+U-Net, TransUNet, EfficientNet-B3, Swin-UNet, MedT, UNETR, and CoTr were trained and evaluated using the same patient-disjoint CBIS-DDSM and RTM data partitions, preprocessing pipeline, augmentation policy, and evaluation metrics adopted for the proposed model. The training, validation, and testing cohorts were therefore identical across the compared architectures. For MSF-Swin-ICFNet, the final mammogram-level classification ROC-AUC values were 0.985 on CBIS-DDSM and 0.992 on RTM, while the corresponding lesion-localization Dice scores were 0.786 and 0.878, respectively. These results indicate high classification discrimination within the evaluated patient-disjoint cohorts, while the lower Dice score on CBIS-DDSM indicates comparatively more challenging lesion localization. Consequently, the comparison demonstrates the joint classification–localization capability of the proposed framework rather than universal superiority across every segmentation endpoint. All reimplemented models were trained using the same patient-disjoint cohorts and common experimental protocol; consequently, the comparative results reported in Table 11 and Table 14 were generated under consistent data-processing and evaluation conditions.

The controlled comparison therefore indicates a trade-off rather than uniform dominance across all evaluated endpoints. The proposed framework provides a common image-derived representation for both classification and lesion localization, but several dedicated segmentation architectures achieve higher overlap scores under particular conditions. This observation is especially relevant for CBIS-DDSM, where the lightweight localization pathway does not outperform all segmentation-specific comparator architectures. Conversely, the classification results indicate that adaptive hierarchical cross-scale fusion provides a strong representation for benign–malignant discrimination within the evaluated cohorts.

**Figure 15:**
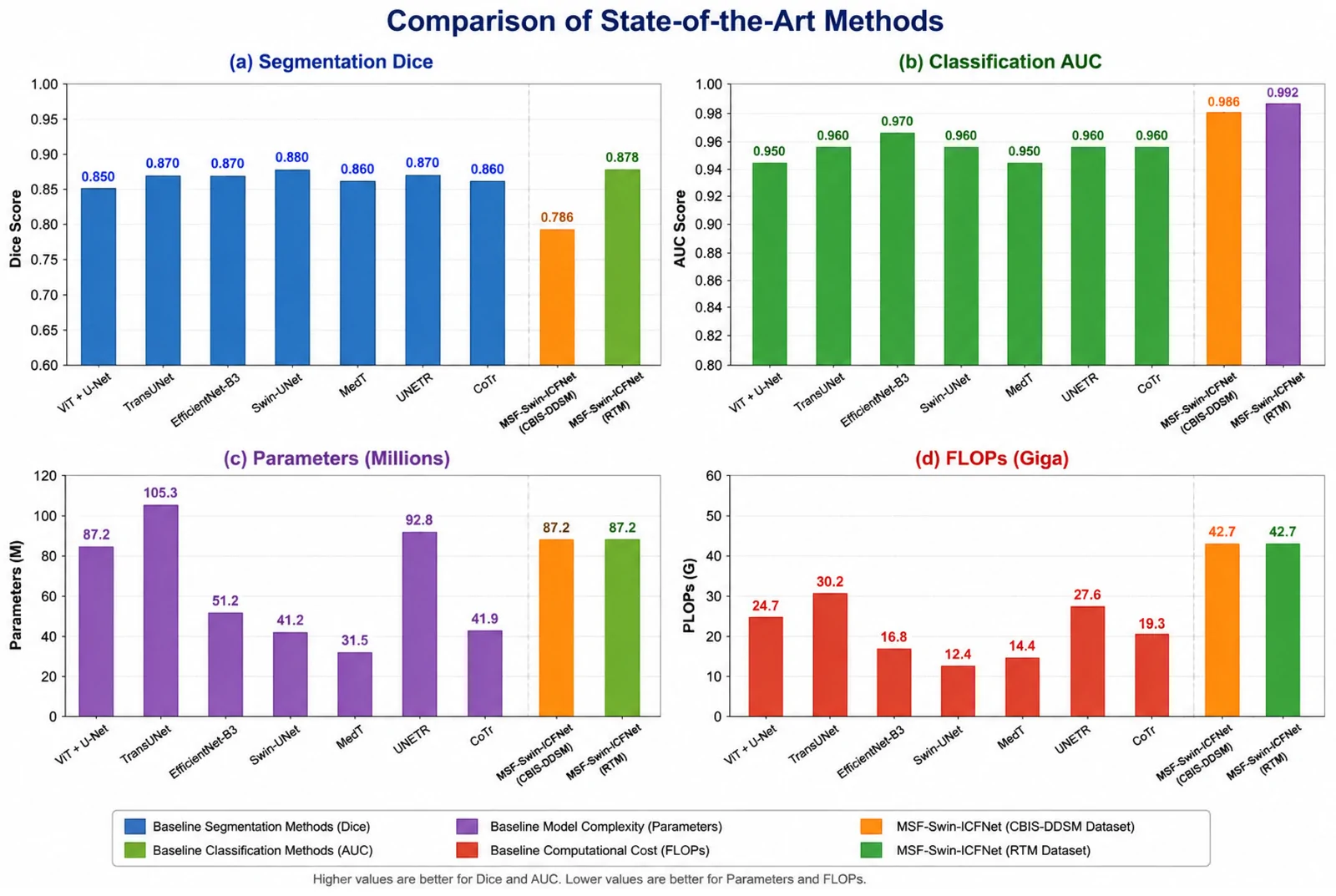
Performance Comparison of MSF-Swin-ICFNet with Existing Transformer and Hybrid Models. ViT: Vision Transformer, MedT: Medical Transformer, UNETR: NEt Transformer, CoTr: Convolutional Transformer.

These results support the usefulness of the proposed architecture as a joint classification–localization framework while also identifying lesion-boundary delineation as an area requiring further improvement. Figure 15 therefore illustrates the comparative trade-off between classification discrimination and segmentation performance rather than evidence of universal superiority of the proposed method. Because all models were evaluated using identical patient-disjoint cohorts, preprocessing procedures, and evaluation metrics, the comparison reduces confounding associated with differences in dataset partitioning and experimental conditions. The observed performance differences therefore provide a more controlled assessment of the contribution of the proposed image-derived cross-scale fusion and lightweight lesion-localization strategy. Figure 15 compares the proposed MSF-Swin-ICFNet model with existing transformer-based and hybrid models using segmentation Dice score and classification AUC. The proposed model achieved a Dice score of 0.786 and ROC-AUC of 0.985 on CBIS-DDSM, and a Dice score of 0.878 and ROC-AUC of 0.992 on RTM. These results indicate strong classification performance across both datasets, while the lower Dice score on CBIS-DDSM suggests more challenging segmentation conditions.

Table 14 provides a controlled comparison of MSF-Swin-ICFNet with representative reimplemented transformer, hybrid, and deep-learning architectures under common patient-disjoint data partitions and evaluation conditions. The comparison reveals different behaviour for classification and segmentation. MSF-Swin-ICFNet achieved strong classification discrimination relative to the evaluated comparator architectures, whereas its segmentation performance was not uniformly superior across all methods and datasets. In particular, the CBIS-DDSM results indicate that the proposed lightweight localization pathway remains sensitive to challenging lesion boundaries and dataset-specific annotation characteristics, while the RTM results show more competitive segmentation behaviour.

These findings suggest that the principal contribution of MSF-Swin-ICFNet lies in obtaining a shared hierarchical representation that supports both benign–malignant classification and lightweight lesion localization rather than in claiming universal superiority for pixel-level segmentation. Cross-dataset differences should also be interpreted cautiously because CBIS-DDSM and RTM differ in acquisition environment, lesion characteristics, image properties, and annotation provenance. The available data do not permit these influences to be isolated individually; therefore, the comparative results are interpreted as performance under the present controlled experimental protocol rather than as evidence of identical behaviour across external clinical populations.

The proposed model achieved a Dice score of 0.786 and ROC-AUC of 0.985 on CBIS-DDSM, and a Dice score of 0.878 and ROC-AUC of 0.992 on RTM. These results indicate strong classification performance across both datasets, while the lower Dice score on CBIS-DDSM suggests comparatively more challenging lesion-localization conditions. The comparison should therefore be interpreted as a trade-off between classification discrimination, lesion localization, and computational complexity rather than as evidence of universal superiority across all endpoints.

### 6.10. Discussions and Limitations

There are several potential sources of dataset bias that should be considered when interpreting the present results. First, CBIS-DDSM and RTM represent different data-acquisition environments. CBIS-DDSM is a curated public benchmark, whereas RTM is a retrospectively collected clinical mammography cohort. Differences in acquisition systems, image quality, lesion prevalence, preprocessing characteristics, and annotation practices may introduce domain or acquisition bias. Because sufficiently detailed and harmonized equipment- and acquisition-level information was not available across the evaluated cohorts, the individual contribution of scanner, detector, acquisition-protocol, and image-processing differences could not be quantified in the present study. Although a standardized preprocessing and normalization strategy was applied, these procedures cannot completely remove variability originating from different imaging environments. Therefore, the performance observed on the evaluated datasets may not directly generalize to mammograms obtained using different imaging systems, acquisition protocols, or clinical institutions.

Second, population and demographic bias cannot be fully assessed, particularly for the RTM cohort. Detailed demographic characteristics, including age distribution, ethnicity, menopausal status, and breast-density distribution, were not available for subgroup analysis. Therefore, the current results do not establish whether model performance is consistent across different demographic or breast-composition groups. The higher segmentation performance observed on RTM relative to CBIS-DDSM could not be attributed to a specific source because lesion morphology, lesion size, breast density, image-quality characteristics, and annotation variability were not systematically matched or stratified across the two datasets. Therefore, future cross-dataset analyses should examine these factors explicitly before drawing conclusions regarding dataset-specific segmentation difficulty or model generalizability.

Third, potential selection and annotation bias should be considered for RTM segmentation. Although the complete RTM dataset contained 10,070 mammograms, lesion masks were available for only 4863 images (48.3%). Consequently, the mask-bearing subset used for segmentation may not fully represent the lesion characteristics and difficulty distribution of the complete RTM cohort. In addition, the original annotation protocol, number of annotators, and their level of expertise were not available for independent verification. The RTM masks were therefore treated as provider-supplied reference annotations rather than independently verified clinical ground-truth delineations.

Fourth, multiple mammograms may originate from the same patient, introducing within-patient correlation among image-level observations. To minimize information leakage, all mammograms belonging to an individual patient were retained within a single training, validation, or testing partition. Nevertheless, the primary performance estimates reported in the manuscript are mammogram-level measures and should not be interpreted as statistically independent patient observations. Complementary patient-level analyses are provided in the Supplementary Materials.

An important limitation of the present study is the absence of fully independent multicentre external validation. Although the evaluation included both the public CBIS-DDSM benchmark and the retrospectively collected RTM clinical mammography cohort, these datasets were incorporated within the model-development and evaluation framework and therefore do not constitute independent multicentre external validation. Consequently, the reported results primarily demonstrate performance within the characteristics of the evaluated cohorts and should not be interpreted as evidence that the model will maintain the same performance across unseen clinical institutions. Variations in mammography systems, acquisition protocols, image quality, patient demographics, breast-density distributions, disease prevalence, and annotation practices may influence model behaviour when deployed in a new clinical environment.

This limitation is particularly important when considering the potential clinical applicability of an AI-based mammography system. Before clinical translation, the finalized model should be frozen and evaluated without retraining, fine-tuning, or threshold optimization on independent patient cohorts obtained from institutions that were not involved in model development. Such external validation should include heterogeneous imaging systems and patient populations and should assess both mammogram-level and patient-level classification performance, together with lesion-localization performance where reliable reference annotations are available. Prospective multicentre studies and radiologist reader evaluations would provide further evidence regarding the robustness, generalizability, and potential clinical utility of the proposed framework. Therefore, the current findings should be regarded as promising retrospective evidence rather than definitive evidence of clinical generalizability. Clinical translation also requires evaluation beyond discrimination metrics alone. A high AUC does not establish an appropriate clinical operating threshold or demonstrate benefit to radiologist decision-making. Future external studies should therefore assess calibration, clinically relevant sensitivity–specificity trade-offs, and performance across lesion and breast-density subgroups whenever reliable metadata are available. Radiologist reader studies should determine whether the classification probability and lesion-localization output improve sensitivity, specificity, interpretation time, or diagnostic confidence relative to unaided assessment. Attention should also be given to discordant cases in which a high malignancy probability is associated with weak or spatially implausible localization. Consequently, the present model should be regarded as a research-stage decision-support framework until reproducible clinical benefit is demonstrated in independent prospective settings.

Future multicentre validation should assess both mammogram-level and patient-level performance in independent clinical populations. Of the 10,070 RTM mammograms, 4863 images (48.3%) were accompanied by provider-supplied lesion masks. The complete RTM cohort contained 10,070 mammograms and was eligible for image-level classification. Of these, 4863 mammograms (48.3%) had corresponding provider-supplied lesion masks and were eligible for segmentation analysis. Patient-level partitioning was performed before constructing the mask-bearing segmentation subsets, and each mammogram and corresponding mask inherited the partition assignment of its source patient. Consequently, no image–mask pair belonging to one patient could appear in more than one partition. RTM mammograms without lesion masks contributed to classification analysis but did not contribute to the segmentation loss or segmentation evaluation. Additional information regarding the annotation procedure and the number and experience of the mask annotators was not available in the released documentation. The RTM masks were therefore treated as dataset-provided reference annotations rather than independently verified provider-supplied reference truth annotations. In addition, detailed RTM demographic information and institution-specific provenance were not available for subgroup analysis, and the original mask-annotation protocol and annotator qualifications could not be independently verified.

Although the study included the clinically acquired RTM cohort in addition to the public CBIS-DDSM benchmark, RTM was not used as a purely external validation cohort because patient-disjoint RTM subsets were used during model development and evaluation. Therefore, the present results should not be interpreted as independent external clinical validation. A rigorous external validation study would require freezing the final model after development on the source cohort and evaluating it, without retraining, fine-tuning, threshold optimization, or model selection, on mammograms obtained from an entirely independent institution. Such multicentre external validation remains an important direction for future work.

## 7. Conclusions

This study presented MSF-Swin-ICFNet, an image-derived multi-scale Swin Transformer framework for benign–malignant breast lesion classification and lightweight lesion localization from mammograms. The framework integrates hierarchical representations from all four Swin Transformer stages through explicit feature alignment, adaptive cross-scale recalibration, and weighted fusion, with the resulting shared representation supporting both classification and lesion-mask prediction. Evaluation on CBIS-DDSM and RTM demonstrated strong retrospective classification and segmentation performance, while the component-wise ablation analysis supported the contribution of cross-scale fusion, feature recalibration, refinement, and the boundary-aware localization pathway. The findings should nevertheless be interpreted within the retrospective experimental setting of the present study. Although patient-level separation was used to reduce information leakage, the evaluated datasets were incorporated within the model-development and evaluation framework and therefore do not provide independent multicentre external validation. The predicted lesion masks and attribution maps may provide complementary spatial information for examining model decisions, but they should not be interpreted as substitutes for radiologist assessment, BI-RADS evaluation, or pathology-based diagnosis. Accordingly, the present results demonstrate the technical potential of the proposed image-derived framework rather than established clinical utility or readiness for clinical deployment.

### Future Work

Future work will prioritize evaluation of a frozen MSF-Swin-ICFNet model on independent multicentre mammography cohorts, including heterogeneous acquisition systems and patient populations. Prospective radiologist reader studies will also be important for determining whether the lesion-localization and classification outputs provide measurable benefit during image interpretation. Additional work will investigate robustness across mammography vendors, domain-shift mitigation, and computationally efficient implementations suitable for broader deployment settings. Because the present study specifically evaluates an image-derived framework, any incorporation of clinical variables will be examined separately through controlled image-only versus image–clinical comparisons using identical patient partitions and standardized clinical information, rather than being treated as part of the current model.

## Figures and Tables

**Table 1:** Distinction between the previous SwinCAMF-Net study and the present framework in the manuscript.

Aspect	Diagnostics-2025 [23]	Present Work
Main design	Multimodal cross-attention fusion	Image-derived cross-scale fusion
Input sources	Mammographic views, 3D ROI/volume features, clinical metadata	Mammographic image-derived feature maps only
Clinical metadata	Used through clinical projection module	Not used
Volumetric/3D ROI input	Used through 3D ROI/volume encoder	Not used
Fusion mechanism	Cross-attention fusion for multimodal alignment	Cross-scale fusion for multi-resolution image feature integration
Segmentation pathway	Multimodal segmentation decoder	Lightweight image-derived mask head
Main novelty	Multimodal explainable fusion	Image-only cross-scale feature representation and lightweight localization
Intended setting	Availability of image, volume, and clinical information	Mammography-only decision-support setting

ROI: Region of Interest.

**Table 2:** Critical comparison of representative mammographic and medical-image analysis strategies.

Approach	Main Strength	Principal Limitation	Differences from MSF-Swin-ICFNet
CNN/U-Net	Preserves texture and boundaries	Limited long-range context; full decoder required	Uses hierarchical Swin context with lightweight localization
Pure Swin/Transformer	Strong contextual modelling	Fine details may be weakened during downsampling	Explicitly aligns and recalibrates multiple stages
Hybrid CNN–Transformer	Combines local and global information	Parallel branches and fixed fusion may add complexity	Does not require a separate CNN encoder
Multimodal image–clinical	Uses complementary patient information	Depends on complete and standardized metadata	Provides an image-only alternative
Multi-scale/attention fusion	Selected multi-level features with attention or direct aggregation	May use selected scales or a segmentation-specific decoder	All four Swin stages are aligned, adaptively recalibrated, and fused into one representation shared by classification and localization
MSF-Swin-ICFNet	Adaptive four-stage image-derived fusion	Requires external patient-level validation	Joint classification and localization from a shared fused representation

CNN: Convolutional Neural Network.

**Table 3:** Characteristics of the Mammography Datasets Used for Model Development and Evaluation.

Dataset	Patients	Benign Patients	Malignant Patients	Mammograms
CBIS-DDSM Mass	891	431	460	1592
RTM	573	307	266	10,070

RTM: Real-Time Mammogram dataset, CBIS-DDSM: Curated Breast Imaging Subset of the Digital Database for Screening Mammography.

**Table 4:** Dataset Characteristics and Patient-Level Partitioning Used in the Present Study.

Characteristic	CBIS-DDSM	RTM
Unique patients	891	573
Benign patients	431	307
Malignant patients	460	266
Full mammograms	1592	10,070
Benign mammograms	Not separately reported	5374
Malignant mammograms	Not separately reported	4696
Lesion annotations/masks	1696 annotated mass regions	4863 mask-bearing mammograms
Training patients	624	401
Validation patients	133	86
Test patients	134	86
Training mammograms	1114	7083
Validation mammograms	239	1498
Test mammograms	239	1489
Split strategy	Stratified patient-level 70:15:15	Stratified patient-level 70:15:15
Random seed	42	42

**Table 5:** Augmentation details.

Augmentation Type	Parameters	Purpose
Geometric Transformation	Rotation ±15°, horizontal flip, translation ±10%, scaling 0.9–1.1	Improve model generalization to positional variations
Intensity Transformation	Brightness/contrast ±10%, gamma correction 0.8–1.2	Simulate variation in mammographic acquisition
Noise and Blur	Gaussian noise σ = 0.01, Gaussian blur σ = 0.5	Improve robustness against imaging artifacts
Mixup Augmentation	Beta distribution with α = 0.2	Improve regularization and reduce overfitting
Lesion-aware Augmentation	Reference lesion masks used to identify lesion extent; training samples containing small or under-represented lesions preferentially selected; identical geometric transformation applied to image–mask pairs	Increase exposure to difficult/small lesion patterns while preserving image–mask spatial correspondence

**Table 6:** Detailed layer-wise architecture and hyperparameters of MSF-Swin-ICFNet.

Component	Hyperparameter	Value
Input	Mammogram size	512 × 512 × 1
Patch embedding	Patch/kernel size	4 × 4
Patch embedding	Stride	4
Patch embedding	Embedding dimension	96
Swin Stage 1	Output resolution/channels	128 × 128 × 96
Swin Stage 1	Number of blocks	2
Swin Stage 1	Attention heads	3
Swin Stage 2	Output resolution/channels	64 × 64 × 192
Swin Stage 2	Number of blocks	2
Swin Stage 2	Attention heads	6
Swin Stage 3	Output resolution/channels	32 × 32 × 384
Swin Stage 3	Number of blocks	6
Swin Stage 3	Attention heads	12
Swin Stage 4	Output resolution/channels	16 × 16 × 768
Swin Stage 4	Number of blocks	2
Swin Stage 4	Attention heads	24
Swin attention	Window size	7 × 7
Swin block	MLP expansion ratio	4
Swin block	Dropout	0
Swin block	Attention dropout	0
Swin block	Drop path	0.2
ICF projection	Projection operation	1 × 1 convolution
ICF alignment	Common spatial resolution	128 × 128
ICF alignment	Common channel dimension (C_f)	96
ICF weighting	Scale-weight normalization	Softmax across four scales
Classification head	Pooling	Global average pooling
Classification head	Output	Fully connected layer + sigmoid
Mask head	Convolution type	2D convolution
Mask head	Refinement blocks	2
Mask head	Kernel size	3 × 3
Mask head	Channels per refinement block	96 → 48
Mask head	Upsampling	2 × bilinear upsampling + 3 × 3 convolution
Mask output	Output layer	1-channel prediction + sigmoid
Mask output	Output size	512 × 512 × 1

**Table 7:** Segmentation Strategy Used in the Proposed Framework.

Step	Method	Purpose
Image encoder	Swin Transformer feature pyramid	Extract hierarchical mammographic features
Cross-scale fusion	Image-Derived cross-scale fusion module	Integrate low-level boundary cues and high-level semantic information
Mask head	Lightweight convolutional upsampling head	Generate lesion localization mask
Boundary refinement	Boundary-aware feature refinement	Improve lesion margin localization

**Table 8:** Hardware and Computational Configuration.

Parameter	Value
Training GPU	NVIDIA A100 40 GB
Batch Size	8
Training Time per Epoch	10.8 min
Total Training Time	7.2 h
Peak Memory Usage	31.9 GB
Training Speed	25.1 samples/s
Inference Time	0.26 s/sample
Throughput	13,846 samples/h
Inference Memory Usage	5.6 GB
FLOPs per Inference	42.7 GFLOPs
Total Parameters	87.2 M
Trainable Parameters	84.6 M
Model Size	312.7 MB
Feature Dimension	1024

GPU = Graphics Processing Unit, FLOP = Floating-Point Operation, GFLOPS = Giga Floating-Point Operations Per Second.

**Table 9:** Segmentation Performance of MSF-Swin-ICFNet on CBIS-DDSM and RTM Datasets with 95% Confidence Intervals.

Dataset	Dice	IoU	Precision	Recall	Hausdorff Distance (mm)
CBIS-DDSM	0.786 ± 0.041 (95% CI: 0.779–0.793)	0.654 ± 0.053 (95% CI: 0.645–0.663)	0.822 ± 0.039 (95% CI: 0.815–0.829)	0.819 ± 0.035 (95% CI: 0.813–0.825)	3.24 ± 0.89 (95% CI: 3.09–3.39)
RTM	0.878 ± 0.046 (95% CI: 0.870–0.886)	0.791 ± 0.052 (95% CI: 0.782–0.800)	0.860 ± 0.041 (95% CI: 0.853–0.867)	0.914 ± 0.043 (95% CI: 0.907–0.921)	2.96 ± 0.88 (95% CI: 2.81–3.11)

Note: Values are reported as mean ± standard deviation. The two-sided 95% confidence intervals were obtained using patient-clustered bootstrap resampling with 1000 repetitions and the percentile method (2.5th and 97.5th percentiles). Segmentation evaluation was restricted to mammograms with available lesion masks, and all mask-bearing mammograms from the same patient were retained within the same bootstrap resample.

**Table 10:** Mammogram-Level Classification Performance of MSF-Swin-ICFNet on Patient-Disjoint CBIS-DDSM and RTM Test Cohorts.

Dataset	Accuracy	Precision	Recall	Specificity	F1-Score	AUC-ROC	AUC-PR
CBIS-DDSM (Test)	0.949	0.944	0.919	0.971	0.931	0.985	0.949
RTM (Test)	0.968	0.956	0.961	0.976	0.956	0.992	0.970

AUC = Area Under the Curve, ROC = Receiver Operating Characteristic, **PR** = **Precision–Recall**, **AUC-PR** = **Area Under the Precision–Recall Curve**. Note: For five-fold cross-validation, the reported sample size refers to the total number of images used across all folds. For the independent test evaluation, the reported sample size refers to the held-out test subset.

**Table 11:** F1-score comparison of MSF-Swin-ICFNet with representative deep-learning models and the previously published SwinCAMF-Net.

Model	F1-Score (CBIS-DDSM)	F1-Score (RTM)
ResNet	0.678	0.748
DenseNet	0.721	0.789
Vision Transformer	0.601	0.694
Medical Transformer	0.658	0.731
Swin-T v2	0.756	0.818
SwinCAMF-Net	0.902	0.944
MSF-Swin-ICFNet Proposed	0.931	0.958

Note: SwinCAMF-Net results are reproduced from Narayanam et al. [23], Diagnostics 2025, 15, 3037, under the Creative Commons Attribution (CC BY 4.0) license with appropriate attribution.

**Table 12:** Cross-Validation Performance of MSF-Swin-ICFNet on CBIS-DDSM and RTM Datasets.

Metric	CBIS-DDSM Mean ± Std. Dev.	95% Confidence Interval	RTM Mean ± Std. Dev.	95% Confidence Interval
Accuracy	0.881 ± 0.012	0.854–0.891	0.976 ± 0.014	0.963–0.988
Precision	0.864 ± 0.019	0.825–0.882	0.959 ± 0.021	0.940–0.988
Recall	0.867 ± 0.018	0.826–0.883	0.972 ± 0.021	0.951–0.997
F1-Score	0.858 ± 0.012	0.823–0.860	0.965 ± 0.013	0.953–0.981
AUC-ROC	0.931 ± 0.015	0.898–0.945	0.989 ± 0.009	0.976–0.998
AUC-PR	0.907 ± 0.011	0.878–0.911	0.995 ± 0.011	0.978–0.999

**Table 13:** Ablation Study Results of MSF-Swin-ICFNet on CBIS-DDSM and RTM Datasets.

Configuration	Accuracy CBIS	F1-Score CBIS	AUC-ROC CBIS	Dice CBIS	Drop CBIS	Accuracy RTM	F1-Score RTM	AUC-ROC RTM	Dice RTM	Drop RTM
Full MSF-Swin-ICFNet	0.949	0.931	0.985	0.786	Baseline	0.968	0.956	0.992	0.878	Baseline
Without Image-Derived Cross-Scale Fusion	0.908	0.872	0.946	0.742	Acc: −4.3%, F1: −6.3%, AUC: −4.0%, Dice: −5.6%	0.914	0.898	0.970	0.846	Acc: −5.6%, F1: −6.1%, AUC: −2.0%, Dice: −3.6%
Without Cross-Scale Recalibration	0.883	0.843	0.921	0.716	Acc: −7.0%, F1: −9.5%, AUC: −6.5%, Dice: −8.9%	0.903	0.889	0.961	0.823	Acc: −6.7%, F1: −7.0%, AUC: −3.1%, Dice: −6.3%
Without Feature-Stack Refinement	0.828	0.790	0.863	0.664	Acc: −12.8%, F1: −15.1%, AUC: −12.4%, Dice: −15.5%	0.855	0.752	0.801	0.759	Acc: −11.7%, F1: −21.3%, AUC: −19.3%, Dice: −13.6%
Without Boundary-Aware Mask Head	0.862	0.823	0.899	0.693	Acc: −9.2%, F1: −11.6%, AUC: −8.7%, Dice: −11.8%	0.871	0.757	0.837	0.788	Acc: −10.0%, F1: −20.8%, AUC: −15.6%, Dice: −10.3%
Swin Encoder Only	0.815	0.770	0.841	0.620	Acc: −14.1%, F1: −17.3%, AUC: −14.6%, Dice: −21.1%	0.826	0.704	0.781	0.716	Acc: −14.7%, F1: −26.4%, AUC: −21.3%, Dice: −18.5%

CBIS: Stands for CBIS_DDSM Dataset.

**Table 14:** Comparison of MSF-Swin-ICFNet with representative Transformer and hybrid models, including the previously published SwinCAMF-Net.

Method	Segmentation Dice	Classification AUC	Parameters (M)	FLOPs (G)
ViT + U-Net	0.850	0.950	86.4	24.7
TransUNet	0.870	0.960	105.3	30.2
EfficientNet-B3	0.870	0.970	51.2	16.8
Swin-UNet	0.880	0.960	41.2	12.4
MedT	0.860	0.950	31.5	14.4
UNETR	0.870	0.960	92.8	27.6
CoTr	0.860	0.960	41.9	19.3
SwinCAMF-Net (CBIS-DDSM)	0.795	0.957	87.2	42.7
SwinCAMF-Net (RTM)	0.931	0.977	87.2	31.2
MSF-Swin-ICFNet (CBIS-DDSM dataset)	0.786	0.985	87.2	42.7
MSF-Swin-ICFNet (RTM dataset)	0.878	0.992	87.2	42.7

Note: The SwinCAMF-Net results are reproduced from Narayanam et al. [23], Diagnostics 2025, 15, 3037, Table 14, under the Creative Commons Attribution (CC BY 4.0) license with appropriate attribution. The remaining comparator models were reimplemented and evaluated under the common patient-disjoint experimental protocol described in this study. Their parameter counts and FLOPs were measured using the corresponding implementations under the same input resolution and computational profiling procedure. The MSF-Swin-ICFNet values correspond to the final implementation reported in Table 7. The previously published SwinCAMF-Net results were not re-evaluated under the present protocol and are therefore included for contextual comparison.

## Data Availability

The CBIS-DDSM dataset utilized in this study is publicly available through The Cancer Imaging Archive (TCIA) repository and can be accessed at Lee, R. S., Gimenez, F., Hoogi, A., and Rubin, D. (2016). *Curated Breast Imaging Subset of DDSM* [2]. The Cancer Imaging Archive. https://doi.org/10.7937/K9/TCIA.2016.7O02S9CY (accessed on 26 May 2026) A minimal anonymized subset of the RTM mammography dataset used in this study has been made publicly available through Figshare at https://doi.org/10.6084/m9.figshare.32314323 (accessed on 26 May 2026).

## Supplementary Materials

The following supporting information can be downloaded at: https://www.mdpi.com/article/10.3390/tomography12090130/s1: Supplementary Tables S1–S9 and Supplementary Figures S1–S5.
