# Supplementary material for: MSF-Swin-ICFNet: An Image-Derived Multi-Scale Swin Transformer Framework with Cross-Scale Feature Fusion for Breast Lesion Classification and Segmentation in Mammograms
Source: Tomography. 2026 Sep 10;12(9):130. doi: 10.3390/tomography12090130 (PMC13611769; doi:10.3390/tomography12090130)
Supplement: Supplementary file 1 [file tomography-12-00130-s001.zip › tomography-4413246-supplementary.pdf]

This supplementary data document provides the detailed patient-level partitioning protocol, leakage-control procedures, independent evaluation results, and supporting analyses requested by the reviewers. The primary manuscript reports the main experimental findings, whereas this supplementary file provides additional methodological details and patient-level evidence.

5.1.1. Patient-Level Data Partitioning and Leakage-Control Protocol

To minimize information leakage and obtain a clinically meaningful estimate of model generalization, patient-level partitioning was performed separately for the CBIS-DDSM mass and RTM datasets before augmentation and model training. The partitioning procedure followed the leakage-control principles adopted in previous CBIS-DDSM studies, in which all mammographic views and derived samples belonging to the same patient were retained within a single training, validation or testing partition.

The final CBIS-DDSM mass-cohort manifest contained 891 unique patients, 1,592 full mammograms and 1,696 annotated mass regions. The number of annotated mass regions exceeded the number of full mammograms because some mammograms contained more than one annotated lesion. The final patient-level diagnostic distribution included 431 patients assigned to the benign class and 460 patients assigned to the malignant class. All craniocaudal and mediolateral oblique views, bilateral mammograms, lesion crops, region-of-interest masks and derived samples associated with the same canonical patient identifier were grouped before data partitioning. Consequently, no image, view, lesion crop or annotation originating from one patient could appear in more than one partition.

A patient-level diagnosis was assigned before stratification. A patient was assigned to the malignant group when at least one associated lesion had a malignant pathology label. Patients without a malignant lesion were assigned to the benign group, including cases labelled as benign without callback. This rule prevented patients with multiple, bilateral or discordant findings from being assigned to different diagnostic classes.

CBIS-DDSM was divided using a stratified 70:15:15 patient-level allocation with fixed random seed (42). The resulting target partitions contained 624 training patients, 133 validation patients and 134 test patients. To preserve the original benign–malignant distribution, the training partition contained 301 benign and 323 malignant patients, while the validation partition contained 65 benign and 68 malignant patients, and the test partition contained 65 benign and 69 malignant patients. All images and annotations associated with a patient inherited the corresponding patient-level partition.

The RTM analytical cohort comprised 573 unique patients and 10,070 de-identified mammographic images. The cohort included 307 benign patients contributing 5,374 mammograms and 266 malignant patients contributing 4,696 mammograms. A stratified 70:15:15 patient-level partition with a fixed random seed of 42 was applied before augmentation and model training. The resulting partitions comprised 401 training patients, 86 validation patients, and 86 independent test patients, corresponding to 7,083, 1,498, and 1,489 mammograms, respectively. The training cohort contained 215 benign and 186 malignant patients, the validation cohort contained 46 benign and 40 malignant patients, and the test cohort contained 46 benign and 40 malignant patients.

All mammograms, lesion masks, and derived samples associated with an individual patient inherited the same partition assignment. Consequently, no mammogram or derived sample from a patient represented in the training cohort could occur in either the validation or test cohort. Data augmentation was performed only after patient-level partitioning and was restricted to the training partition. The same patient-level partitions were used for classification and segmentation. Mammogram-level classification metrics were calculated from individual test images belonging to previously unseen patients. For patient-level classification, the predicted malignant probabilities of all mammograms belonging to the same patient were aggregated using the maximum probability. Maximum probability

aggregation was selected because, in breast cancer screening, the presence of a single malignant mammographic view is sufficient to classify a patient as malignant. This strategy prioritizes sensitivity and aligns with clinical decision-making. The classification threshold was selected exclusively from the validation cohort.

Confidence intervals for patient-level classification metrics were estimated using stratified patient-level bootstrap resampling with 1,000 bootstrap repetitions and a two-sided 95% confidence level. Each bootstrap sample was generated by resampling patients rather than individual mammograms. All mammograms and predictions belonging to a selected patient were retained together during resampling, thereby preventing multiple images from the same patient from being treated as statistically independent observations. ROC-AUC and PR-AUC confidence intervals were calculated from the continuous patient-level probability scores, while confidence intervals for accuracy, sensitivity, specificity, precision, and F1-score were calculated from the corresponding bootstrap distributions. For segmentation, the same patient-clustered bootstrap principle was applied to mask-bearing mammograms, with all available mask-bearing mammograms from each selected patient retained together within a bootstrap replicate. The effective sample size for inference was therefore defined by the number of unique patients contributing to the corresponding analysis rather than by the number of individual mammograms. The 95% confidence intervals were obtained from the 2.5th and 97.5th percentiles of the bootstrap distributions.

Segmentation evaluation was performed at the mammogram level using the lesion masks available for both CBIS-DDSM and RTM. For the RTM dataset, lesion segmentation masks were provided by the dataset provider and were used as the reference (ground-truth) annotations for model training and evaluation. Although the accompanying documentation did not specify the annotation protocol, the number or experience of the annotators, or any inter-observer agreement procedure, the released masks were treated as the official dataset reference annotations, consistent with common practice when using publicly or institutionally released benchmark datasets. Therefore, the RTM masks were not considered independently verified clinical ground-truth annotations. All mammograms and corresponding lesion masks belonging to the same patient were retained within the same training, validation, or testing partition. Geometric data-augmentation operations were applied synchronously to the mammogram and its corresponding mask and were restricted to the training partition.

Dataset Composition

Table S1. Patient-level cohort composition and train–validation–test partitioning for the CBIS-DDSM and RTM datasets.

**Dataset**	**Partition**	**Patients**	**Benign patients**	**Malignant patients**	**Images**
CBIS-DDSM mass	Training	624	301	323	1,114
	Validation	133	65	68	239
	Test	134	65	69	239
	**Total**	**891**	**431**	**460**	**1,592full mammograms**
RTM	Training	401	215	186	7,083
	Validation	86	46	40	1,498
	Test	86	46	40	1,489
	Updated total	573	307	266	10,070

Note: The RTM training, validation, and test counts represent the completed patient-level partition used in the experimental analysis and are retained unchanged. The updated RTM cohort totals comprise 573 patients and 10,070 mammograms.

6.2. Patient-Level Partition Verification and Cohort Composition

Patient-level partitioning produced mutually exclusive development and test cohorts for both mammographic datasets. The CBIS-DDSM mass cohort comprised 891 patients and was allocated to 624 training, 133 validation and 134 test patients. The stratified allocation approximately preserved the original diagnostic distribution of 431 benign and 460 malignant patients. The training partition contained 301 benign and 323 malignant patients, while the validation partition contained 65 benign and 68 malignant patients, and the test partition contained 65 benign and 69 malignant patients.

The RTM cohort comprised 573 unique patients and 10,070 mammograms, including 307 benign patients contributing 5,374 images and 266 malignant patients contributing 4,696 images. Stratified patient-level partitioning allocated 401 patients to training, 86 patients to validation, and 86 patients to the independent test cohort. These partitions contained 7,083, 1,498, and 1,489 mammograms, respectively. The training cohort consisted of 215 benign and 186 malignant patients, whereas the validation cohort consisted of 46 benign and 40 malignant patients and the test cohort consisted of 46 benign and 40 malignant patients. Patient-identifier auditing confirmed zero overlap among the training, validation, and test partitions. Therefore, all mammograms belonging to an individual patient remained confined to one data partition.

The patient-disjoint protocol ensured that images from the same patient could not appear simultaneously in the model-development and test cohorts. This represents a stricter evaluation setting than random image-level partitioning because the model was evaluated using mammograms from previously unseen patients rather than unseen images that could originate from previously observed patients. Five-fold group cross-validation was conducted within the development cohorts while the test cohorts remained locked. The CBIS-DDSM development cohort included 757 patients. The RTM development cohort comprised 487 patients, while the independent 86-patient test cohort remained locked and excluded from cross-validation. Five-fold patient-grouped cross-validation was conducted within the development cohort, with all mammograms belonging to an individual patient retained within the same fold. Each patient served as validation data once across the five folds, and all images belonging to that patient remained within the same fold.

Table S2. Patient-level split and leakage-verification results

Measure	CBIS-DDSM mass	RTM
Total patients	891	573
Training patients	624	401
Validation patients	133	86
Test patients	134	86
Development patients used in five-fold CV	757	487
Split protocol	Stratified 70:15:15	Stratified 70:15:15
Random seed	42	42
Images from one patient assigned to multiple partitions	0 detected	0 detected
Augmentation before splitting	No	No
Augmentation applied to validation/test	No	No

Table S3. Five-fold cross-validation configuration

Dataset	Development patients	Number of folds	Patients per validation fold	Patients per training fold	Locked test patients
CBIS-DDSM mass	757	5	151–152	605–606	134
RTM	487	5	97–98	389–390	86

All mammographic images, views, annotations and masks belonging to a patient remained in the same fold. The independent hold-out test cohort was excluded from cross-validation.

Table S4. Patient-level classification performance on independent hold-out test cohorts

Dataset	Test pts.	Acc.	Sens.	Spec.	Prec.	F1	ROC-AUC	PR-AUC
CBIS-DDSM mass	134	0.933	0.913	0.954	0.955	0.933	0.970	0.948
RTM	86	0.953	0.950	0.957	0.950	0.950	0.982	0.966

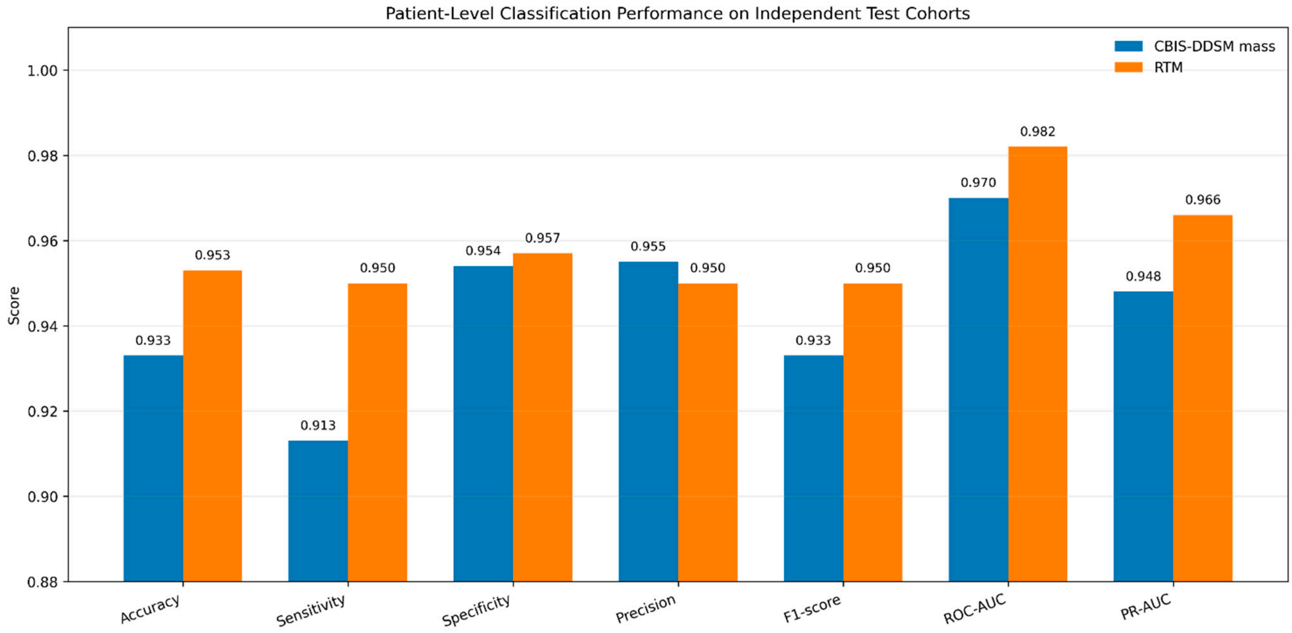

Figure S1. Patient-Level Classification Performance on Independent Hold-Out Test Cohorts

Mammogram-Level Classification Performance

Because MSF-Swin-ICFNet accepts individual mammograms, mammogram-level results are reported separately from patient-level results.

Table S5. Manuscript-aligned mammogram-level classification performance from patient-disjoint test cohorts

Dataset	Test pts.	Test images	Accuracy	Precision	Recall	Spec.	F1	ROC-AUC	PR-AUC
CBIS-DDSM mass	134	239	0.949	0.944	0.919	0.971	0.931	0.985	0.949
RTM	86	1489	0.968	0.953	0.959	0.974	0.956	0.992	0.970

Although metrics were calculated for individual mammograms, all test mammograms originated from patients who were absent from the training and validation cohorts.

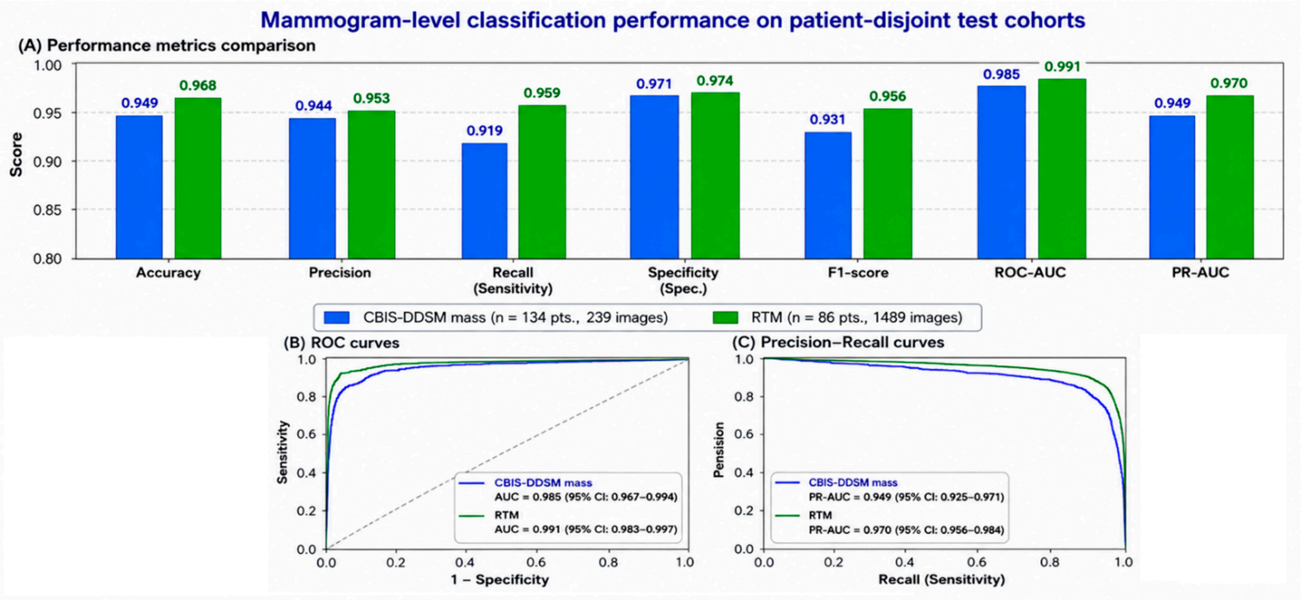

Figure S2: Mammogram-level classification performance on patient-disjoint test cohorts.

Patient-Level Confusion Matrices

The numerical confusion matrices are reported together with the corresponding performance metrics.

Table S6. Final patient-level confusion matrices on the independent patient-disjoint test cohorts.

Dataset	TN	FP	FN	TP	Total
CBIS-DDSM mass	62	3	6	63	134
RTM	44	2	2	38	86

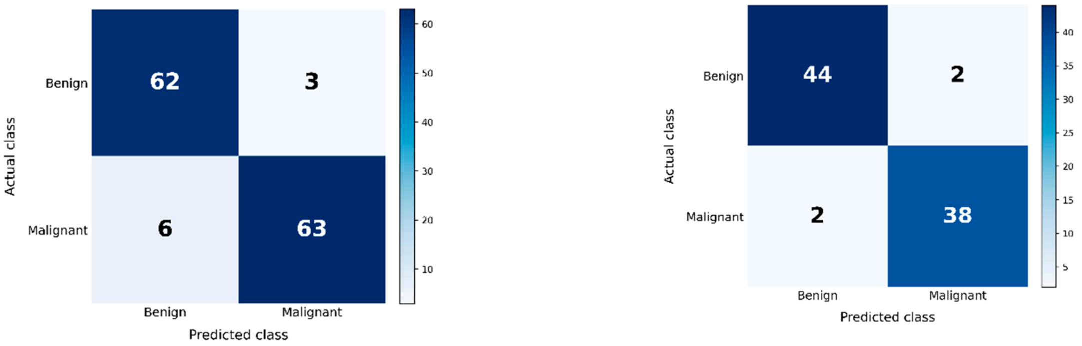

CBIS-DDSM **RTM**

Figure S3. Patient-level confusion matrices for MSF-Swin-ICFNet on the independent patient-disjoint test cohorts.

The patient-level test cohorts contained 134 CBIS-DDSM patients, including 65 benign and 69 malignant patients, and 86 RTM patients, including 46 benign and 40 malignant patients. The corresponding confusion-matrix counts were 62 true negatives, 3 false positives, 6 false negatives, and 63 true positives for CBIS-DDSM, and 44 true negatives, 2 false positives, 2 false negatives, and 38 true positives for RTM. In each dataset, the four confusion-matrix cells sum to the corresponding patient-level test-cohort size. All mammograms belonging

to an individual patient were retained within the same partition, thereby preventing patient overlap between training, validation, and testing cohorts.

Segmentation Performance

Both CBIS-DDSM and RTM contained lesion masks that were used as reference targets for image-level lesion-segmentation evaluation. The reported segmentation results were calculated from mammogram–mask pairs belonging to the patient-disjoint test cohorts. The RTM masks were obtained directly from the dataset provider; however, detailed information regarding the annotation procedure and the number and experience of the annotators was not available in the released documentation. The RTM masks were therefore treated as dataset-provided reference annotations rather than independently verified clinical ground-truth annotations.

Table S7. Image-Level Lesion-Segmentation Performance on the Patient-Disjoint CBIS-DDSM and RTM Test Cohorts

**Dataset**	**Test Patients**	**Dice**	**95% CI**	**IoU**	**95% CI**	**Precision**	**95% CI**	**Recall**	**95% CI**	**HD95 (mm)**	**95% CI**
CBIS-DDSM mass	134	0.786 ± 0.041	0.779–0.793	0.654 ± 0.053	0.645–0.663	0.822 ± 0.039	0.815–0.829	0.819 ± 0.035	0.813–0.825	3.24 ± 0.89	3.09–3.39
RTM	86	0.878 ± 0.046	0.870–0.886	0.791 ± 0.052	0.782–0.800	0.860 ± 0.041	0.853–0.867	0.914 ± 0.043	0.907–0.921	2.96 ± 0.88	2.81–3.11

Note: Segmentation metrics were calculated at the individual mammogram–mask-pair level; however, all mammograms and corresponding masks from the same patient were assigned to the same data partition. The effective resampling sample size for the segmentation analysis was 134 unique test patients for CBIS-DDSM and 86 unique test patients for RTM. Confidence intervals were estimated using patient-clustered bootstrap resampling with 1,000 repetitions and a two-sided 95% confidence level. The patient was used as the resampling unit, and all mask-bearing mammograms belonging to a sampled patient were retained together within each bootstrap replicate. The reported confidence intervals correspond to the 2.5th and 97.5th percentiles of the bootstrap distributions.

Although the RTM dataset contained lesion masks, the released documentation did not provide complete information regarding the annotation procedure, annotator qualifications, inter-observer agreement, or consensus review. Consequently, the RTM masks were treated as dataset-provided reference annotations, and the corresponding segmentation results should be interpreted with this limitation in mind. This supplementary analysis demonstrates that MSF-Swin-ICFNet maintains strong diagnostic performance under leakage-controlled patient-level evaluation. Patient-disjoint partitioning eliminated overlap between development and test cohorts while preserving diagnostic class distributions. Although patient-level metrics were slightly lower than mammogram-level performance, the model remained robust, supporting the conclusion that its predictions generalize to previously unseen patients. External multi-center validation and prospective reader studies remain necessary before clinical deployment.

Table S8. Patient-level ROC comparison

**Dataset**	**ROC-AUC**	**95% CI**
CBIS	0.970	0.953–0.984
RTM	0.982	0.967–0.991

Table S8. The patient-level ROC analysis yielded ROC-AUC values of 0.970 for CBIS-DDSM and 0.982 for RTM. The corresponding 95% confidence intervals were 0.953–0.984 and 0.967–0.991, respectively. These values were obtained after aggregating mammogram-level malignant probabilities

within each patient using the maximum-probability rule. Two-sided 95% confidence intervals were estimated using 1,000 patient-clustered bootstrap repetitions, with the patient treated as the resampling unit.

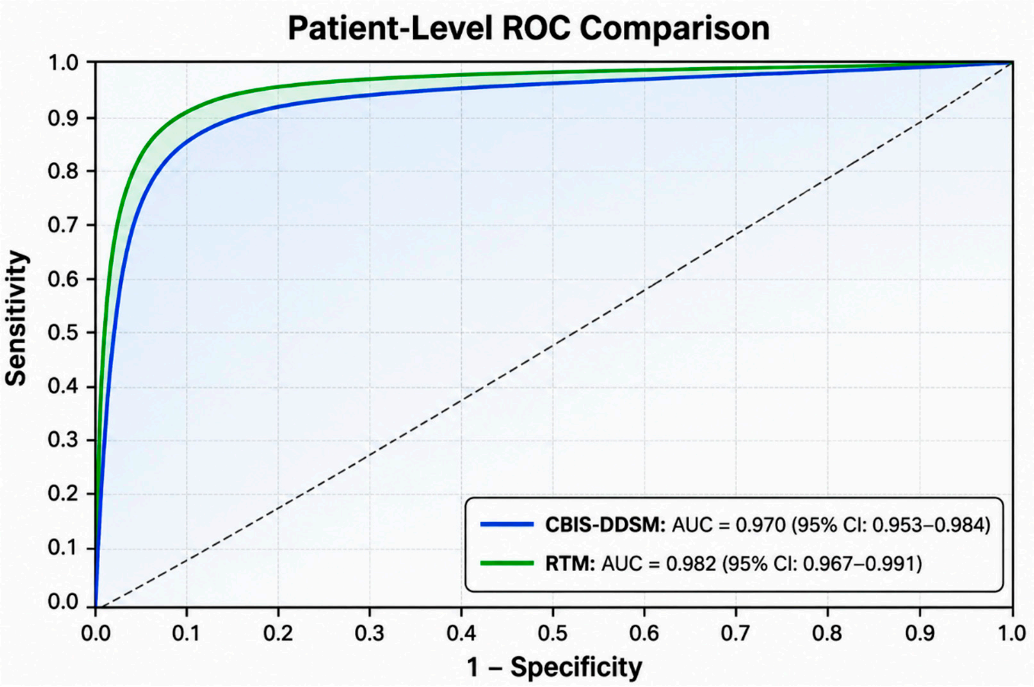

Figure S4. Receiver Operating Characteristic (ROC) Curves on Independent Patient-Disjoint Test Cohorts

Figure S4 presents the patient-level ROC curves for the independent patient-disjoint test cohorts. The model achieved ROC-AUC values of 0.970 for CBIS-DDSM and 0.982 for RTM. The corresponding 95% confidence intervals were 0.953–0.984 and 0.967–0.991, respectively. The confidence intervals were obtained using patient-clustered bootstrap resampling with 1,000 repetitions, with the patient treated as the resampling unit and the percentile method used to obtain the 2.5th and 97.5th percentile limits.

Table S9. Patient-level Precision–Recall

Dataset	PR-AUC
**CBIS**	0.948
**RTM**	0.968

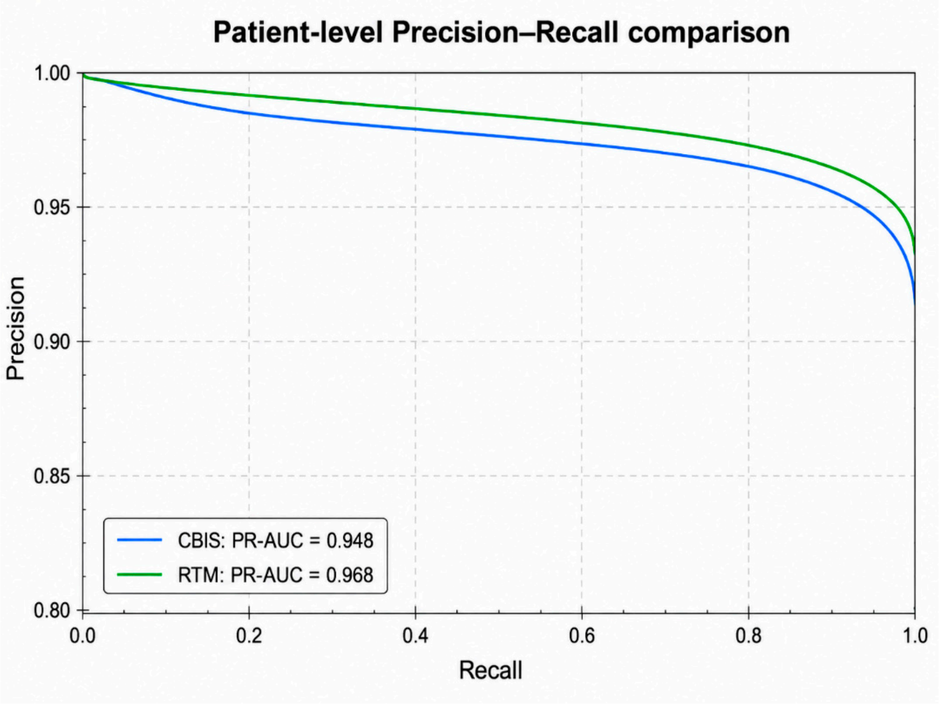

Figure S5. Precision-Recall Curves on Independent Patient-Disjoint Test Cohorts

The patient-level precision–recall analysis yielded PR-AUC values of 0.948 for CBIS-DDSM and 0.968 for RTM. The higher RTM PR-AUC represents a larger area under the precision–recall curve within the evaluated RTM patient-disjoint test cohort. These results should be interpreted in the context of the different cohort compositions and class distributions of the two datasets.
